# Integration of RRBS and RNA-seq unravels the regulatory role of *DNMT3A* in porcine Sertoli cell proliferation

**DOI:** 10.3389/fgene.2023.1302351

**Published:** 2024-01-09

**Authors:** Dong Xu, Saina Yan, Huimin Jin, Chujie Chen, Xiangwei Tang, Xu Wang, Yonghong Li, Fang Fei, Anqi Yang

**Affiliations:** ^1^ Department of Biological and Environmental Engineering, Yueyang Vocational Technical College, Yueyang, Hunan, China; ^2^ School of Basic Medical Sciences, Hengyang Medical School, University of South China, Hengyang, Hunan, China; ^3^ College of Animal Science and Technology, Hunan Agricultural University, Changsha, Hunan, China

**Keywords:** *DNMT3A*, Sertoli cells, proliferation, RRBS, RNA-seq

## Abstract

*DNMT3A* participates in *de novo* methylation, yet its impact on the proliferation of testicular Sertoli cells remains unclear. Development-specific methylation has been proven to be associated with cellular development. Therefore, in this study, we simulated *DNMT3A* expression pattern during testicular development by *DNMT3A* interference. Then, RRBS and RNA-seq were used to decipher *DNMT3A* regulatory mechanisms on Sertoli cell proliferation. Immunofluorescence staining revealed the expression of *DNMT3A* in the Sertoli cells of the prepubertal testis. *DNMT3A* was demonstrated to inhibit the cell cycle and proliferation of Sertoli cells, while promoting cell apoptosis. After transfected with *DNMT3A* interference, a total of 560 DEGs and 2,091 DMGs produced by *DNMT3A* interference were identified between two treated groups, respectively. Integrating the results from RRBS and RNA-seq, the overlapping genes between DMGs and DEGs were found to be enriched in the Gene Ontology (GO) terms related to cellular development and the Apelin signaling pathway. The present study demonstrated the impact of *DNMT3A* on the proliferation of porcine testicular Sertoli cells, suggesting that DNMT3A primarily acts through the Apelin signaling pathway. These findings provide valuable insights into how DNMT3A influences testicular development and health, offering new perspectives.

## 1 Introduction

Sertoli cells play a central role in the mammalian reproductive system, exhibiting essential properties (Griswold, 2018). Notably, they secrete the anti-Müllerian hormone, which inhibits the development of female sexual organs once testicular determination is established during embryogenesis (Wong and Khan, 2022). As the primary cell type within the seminiferous tubules, Sertoli cells are critical for testicular structural integrity and function (Orth et al., 1988). They create a specialized microenvironment for spermatogenesis, including the formation of the blood-testis barrier, which prevents direct contact between maturing germ cells and blood. Moreover, Sertoli cells provide essential nourishment to support germ cell growth and maturation by secreting growth factors and cytokines, which are tightly regulated to ensure proper sperm development (Ni et al., 2019). Studies have shown that Sertoli cell proliferation occurs rapidly after birth and stabilizes with maturity (Zhao et al., 2020). The quantity of Sertoli cells directly correlates with testicular volume and sperm production per ejaculation (Orth et al., 1988). Investigating Sertoli cells is crucial for understanding spermatogenesis mechanisms and gaining insights into testicular development and health.

DNA methylation, a fundamental epigenetic modification, plays a vital role in regulating gene expression without altering the DNA sequence (Moore et al., 2013). It significantly influences various biological phenomena, including development, aging, and memory formation, and is susceptible to environmental factors (Greenberg and Bourchis, 2019). DNA methylation primarily occurs at CpG sites, cytosines preceding guanine nucleotides, and is catalyzed by DNA methyltransferases (DNMTs), including DNMT1, DNMT3A, and DNMT3B (Chen and Zhang, 2020). DNMT1 maintains methylation patterns during cell division, while DNMT3A and DNMT3B are involved in *de novo* methylation during development (Dan and Chen, 2016; Chen and Zhang, 2020). Notably, DNMT3A plays a pivotal role in several biological processes, particularly in reproductive biology (Dura et al., 2022). For example, it has been reported that *DNMT3A* regulates embryonic development (Zhang et al., 2018), stem cell differentiation, and genomic imprinting (Kaneda et al., 2004), all of which have implications for testicular development.

Our previous research has demonstrated the downregulation of *DNMT3A* during testicular maturation, with minimal expression observed in mature testes. Furthermore, we also identified *DNMT3A* as a differentially expressed member within the DNMT family, suggesting its potential involvement in dynamic changes in testicular genome methylation and Sertoli cell proliferation (Anqi et al., 2022). To explore this mechanism, we performed knockdown experiments to reduce *DNMT3A* expression in immature Sertoli cells. The results indicated that *DNMT3A* acts as an inhibitor of proliferation and promotes apoptosis. Subsequent investigations using reduced representation bisulfite sequencing (RRBS) and RNA sequencing (RNA-seq) revealed the impact of *DNMT3A* downregulation on genomic methylation and gene expression patterns. Our integrated analysis suggests that the downregulation of *DNMT3A* influences Sertoli cell proliferation, likely through the modulation of the Apelin signaling pathway and MicroRNAs in cancer. These findings provide novel insights into the regulatory mechanism of *DNMT3A* on Sertoli cell proliferation, further contributing to our understanding of testicular development and health.

## 2 Materials and methods

### 2.1 Ethics statement

All procedures in the present study were conducted in accordance with the Declaration of Helsinki. Sample collection received approval from the Ethics Committee of Yueyang Vocational Technical College (Approval No. 2023–08). Animal welfare was ensured at all stages of the study, preventing unnecessary suffering of the boars.

### 2.2 Testicular sample collection and hematoxylin and eosin staining

Testicular samples were collected from Shaziling boars at the ages of 23 and 110 days, which were designated solely for fattening purposes and not for breeding. Prior to the collection of testicular samples, the boars were administered general anesthesia using Zoletil 50 (Virbac Co., France). The surgical area was then disinfected with medical-grade alcohol, followed by drying with sterile gauze. A 5% iodine tincture (Stary Co., China) was applied for further antiseptic cleansing. An incision was made in the scrotal epidermis, just large enough to extrude the testes. The testicles were then removed via a ligature method. The entire procedure was conducted in a sterile environment. Post-surgery, the boars were housed in clean conditions until full recovery. Standard histological techniques were applied to testis tissue preserved in 4% paraformaldehyde. Light microscopy was utilized to examine the morphological attributes of testicular tissues, following the staining of six-micrometer-thick sections with Masson’s trichrome.

### 2.3 Immunofluorescence staining

Testicular samples, freshly collected from 23-day-old Shaziling boars, were promptly fixed in 4% formaldehyde for 72 h. Sections of these samples were then prepared following standard histological protocols. The slides were incubated for 2 hours at 37°C with either anti-SOX9 (1:3000, Proteintech Group, Chicago, IL, USA, 67439-1-Ig) or anti-DNMT3A (1:200, HuaBio, Hangzhou, China, ST04-78). After an additional hour of incubation at 37°C with secondary antibodies, the sections were rinsed in phosphate-buffered saline (PBS) thrice, for 5 min each time. The testes slices were then treated with DAPI solution (50 ng/mL, Sigma, Louis, MO, USA), and incubated at 26°C for 5 min, followed by another three rinses with PBS. The final images were captured with a Zeiss LSM 510 META confocal laser scanning microscope.

### 2.4 Sertoli cells culture and transfection

An immature porcine Sertoli cell line (ATCCCRL-1746^TM^, AnWei-sci, Shanghai, China) was utilized in the present study to model the expression of the *DNMT3A* gene in Sertoli cells *in vitro*. The immature Sertoli cells were maintained in Dulbecco’s modified Eagle medium (DMEM, Gibco, Grand Island, NY, USA), supplemented with 10% fetal bovine serum (Gibco, Grand Island, USA). The cells were incubated at 37°C in a 5% CO_2_ environment.

Three RNAi oligonucleotides (si-*DNMT3A*-1, -2, -3) were synthesized and provided by RiboBio (Guangzhou, China), with their respective sequences detailed in Supplementary Table S1. Seeding of Sertoli cells was done in a 6-well culture plate, with a cell density of 1 × 10^6^ cells/well, followed by cultivation in a 2 mL medium. Transfection was carried out by diluting 20 uM (achieving a final concentration of 50 nM in the cells) of siRNA or siRNA NC (si-NC) (RiboBio, China) with 120 μL of serum-free Opti-MEM (Thermo Fisher Scientific Inc., Grand Island, NY, USA), with incubation at 25°C for 5 min. Following this, 12 μL of Lipofectamine^TM^ 2000 (Invitrogen, Carlsbad, CA, USA) was also diluted with serum-free Opti-MEM (Gibco, Grand Island, NY, USA), and incubated at 25°C for 30 min. When the cell confluence reached approximately 60%, the resulting mixtures were introduced to each well. After 5 h of incubation at 34°C in a 5% CO_2_ environment, the complete medium was introduced for cultivation. In the present study, Sertoli cells that underwent *DNMT3A* interference were categorized as the treatment group (named as PC), whereas those treated with si-NC were classified as the control group (named as NC). Each group comprised three biological replicates.

### 2.5 Cell cycle assay

A cell cycle analysis kit (Beijing 4A Biotech, Beijing, China) was employed to examine the cell cycle distribution. After 48 h of transfection, Sertoli cells were washed twice with phosphate-buffered saline (PBS) and harvested in a 1.5 mL centrifuge tube. Following a 12-hour incubation at −4°C in 75% (v/v) ethanol, the cells were treated with a propidium iodide (PI) solution (50 mg/mL) for 30 min at 37 °C. Subsequently, the prepared cell suspension was subjected to analysis using an FACSCanto II Flow Cytometer (Becton Dickinson, Franklin Lakes, NJ, USA).

### 2.6 Cell proliferation assay

Cell proliferation was assessed using the Cell Counting Kit-8 (CCK-8) (BioScience, Shanghai, China) and 5-ethynyl-2′-deoxyuridine (EdU) incorporation assays (BioScience, Shanghai, China). Cells were seeded at a density of 5 × 10^3^ cells/well in a 96-well culture plate with 100 μL of culture medium. For the CCK-8 assay, CCK-8 reagent was added to each well after transfection for 24, 48, and 72 h, followed by incubation at 37°C for 4 hours. The absorbance at 450 nm was measured using an enzyme-linked immunosorbent assay (ELISA) plate reader (Thermo Fisher Scientific, Waltham, MA, USA. For the EdU assay, EdU medium was added to each well 48 h post-transfection, and the cells were incubated for 2 h at 37°C. Subsequently, DNA staining solution and EdU staining solution were applied to mark the living cells (blue) and proliferating cells (red), respectively, according to the manufacturer’s protocols. Cell counts were determined using a fluorescence microscope at ×20 magnification and the ImageJ software.

### 2.7 Quantitative real-time PCR (qPCR)

qPCR was conducted following the protocols described in our previous studies. Total RNA was extracted using the TRIzol reagent (Invitrogen, Carlsbad, CA, USA) according to the manufacturer’s instructions. The primers, designed using Oligo 7.0 software (Integrated DNA Technologies, Coralville, IA, USA) (Supplementary Table S2), were synthesized by Sango Bio (Shanghai, China). The cDNA of each sample was synthesized using a cDNA synthesis kit (TaKaRa, Beijing, China). qPCR was performed using a Thermo Scientific PIKO REAL 96 real-time PCR system with the SYBR Green kit (TaKaRa, Beijing, China) as per the manufacturer’s instructions. *GAPDH* was used as the internal control, and each experiment was repeated three times. The gene expression level was normalized to *GAPDH* using the 2^−ΔΔCT^ method.

### 2.8 Cell apoptosis assay

After a 48-hour transfection period, Sertoli cells were collected and transferred to a 1.5-mL centrifuge tube. Subsequently, the cells were washed three times and double-stained using an Annexin V-FITC apoptosis detection kit (Beijing 4A Biotech, Beijing, China). The flow cytometric analysis of cell apoptosis rates was performed using the FACSCanto II Flow Cytometer (Becton Dickinson, Franklin Lakes, NJ, USA). The percentages of cells undergoing early apoptosis and late apoptosis were determined and used to calculate the overall cell apoptosis rate.

### 2.9 Western blotting

After a 48-hour transfection, the total cellular protein was extracted using the radioimmunoprecipitation assay (RIPA) lysis buffer (Bioss, Beijing, China), and the protein concentrations were quantified using the bicinchoninic acid (BCA) protein assay kit (Beyotime, Shanghai, China). The protein samples were subjected to boiling and separated by electrophoresis on 10% SDS-polyacrylamide gels. Subsequently, they were transferred onto a PVDF membrane (Beyotime, Shanghai, China). The membrane, containing protein fractions, was blocked with QuickBlock^TM^ Blocking Buffer (Beyotime, Shanghai, China) for 30 min and then incubated with primary antibodies for 12 h at 4 °C. The primary antibodies included DNMT3A, BAX (1:2000, Proteintech Group, Chicago, IL, USA, 50599-2-Ig), Caspase-3 (1:1000, Cell Signaling Technology, Danvers, MA, USA, 14220), and β-actin (1:5000, Proteintech Group, Chicago, IL, USA, 66009-1-Ig). Following washing, the membrane was incubated with secondary antibodies for 2 h at 26°C. Protein bands were visualized using an ECL advanced Western blotting detection kit (Beyotime, Shanghai, China), with β-actin serving as the loading control.

### 2.10 Adenosine triphosphate (ATP) assay

After transfection for 48 h, Sertoli cells were harvested and transferred to a 1.5-mL centrifuge tube. The ATP concentration was quantified using an ATP assay kit (Beyotime, Shanghai, China) following the manufacturer’s instructions. The relative light units (RLUs) were measured using a Chemiluminescence Apparatus (Thermo Fisher Scientific Inc., Waltham, MA, USA).

### 2.11 RRBS and RNA-seq library construction

After a 48-hour transfection, Sertoli cell RNA and DNA were extracted using TRIzol reagent (Invitrogen, Carlsbad, CA, USA) and Animal Genomic DNA Kit (Tiangen, Beijing, China), respectively. These extractions followed the manufacturers’ protocols and were used to construct libraries for RRBS and RNA-seq analysis, and each group comprised three biological replicates (PC_1, PC_2, PC_3, NC_1, NC_2, and NC_3).

A total of 5.2 µg of genomic DNA, spiked with 26 ng of lambda DNA, was subjected to sonication using a Covaris S220 system (Covaris, Woburn, MA, USA) to fragment the DNA into 200–300 bp fragments. Subsequently, end repair and adenylation were performed. Cytosine-methylated barcodes were ligated to the sonicated DNA following the manufacturer’s instructions. The DNA fragments were then subjected to bisulfite treatment twice using the EZ DNA Methylation-Gold™ Kit (Zymo Research, USA, Catalog #: D5005). The resulting single-stranded DNA fragments were PCR amplified using KAPA HiFi HotStart Uracil + ReadyMix (2X). The quality of the library was assessed on the Agilent 5400 system (Agilent, USA), and quantification was performed by qPCR (1.5 nM). Paired-end sequencing of the sample was conducted on an Illumina Novaseq 6000 platform (Illumina, USA).

Total RNA was isolated using poly-T oligo-attached magnetic beads, followed by treatment with divalent cations at an elevated temperature in First Strand Synthesis Reaction Buffer (×5). The first strand cDNA was synthesized using random hexamer primers and M-MuLV Reverse Transcriptase (RNase H-), followed by synthesis of the second strand cDNA using DNA Polymerase I and RNase H. Subsequently, the DNA fragments underwent adenylation at the 3′ ends and cluster generation. The library was then constructed on an Illumina NovaSeq 6000 platform, generating 150 bp paired-end reads. Both RRBS and RNA-seq libraries were prepared by Novogene Corporation (Beijing, China).

The raw sequence data of RRBS and RNA-seq in the present study were deposited in the Genome Sequence Archive (Genomics, Proteomics and Bioinformatics, 2021), in National Genomics Data Center (Nucleic Acids Res 2022), China National Center for Bioinformation/Beijing Institute of Genomics, and Chinese Academy of Sciences (GSA: CRA011480 and CRA011482). These are publicly accessible at https://ngdc.cncb.ac.cn/ (accessed on 30 June 2023) (Chen et al., 2021; Me and mbers, 2022).

### 2.12 RNA-seq analysis

The raw reads in fastq format were processed using custom Perl scripts. Clean reads were obtained by removing reads with adapters, poly-N content exceeding 10%, and low-quality reads from the raw data. The index of the reference genome (*Sscrofa11.1*) and alignment of paired-end clean reads were performed using HISAT2 v2.0.5 (Mortazavi et al., 2008). The read counts for each gene were determined using featureConts (v1.5.0-p3) (Liao et al., 2014). Fragments per kilobase of exon per million fragments (FPKM) for each gene were calculated using StringTie (v1.3.1) (Pertea et al., 2016).

### 2.13 Identification of differentially expressed genes (DEGs)

Differential expression analysis of the two groups was conducted using the DESeq2 R package (version 1.20.0) with a negative binomial distribution-based model (Anders and Huber, 2010; Young et al., 2010). Genes exhibiting |log2(fold change)| > 1 and an adjusted *p*-value <0.05 were considered as DEGs.

### 2.14 RRBS analysis

The Bismark software (version 0.24.0) (Krueger and Andrews, 2011) was employed to align the raw reads to the *Sscrofa11.1* reference genome. Prior to alignment, the reference genome was converted into a bisulfite-converted version (C-to-T and G-to-A converted) and indexed using bowtie2 (Langmead and Salzberg, 2012). Additionally, the clean reads were fully bisulfite-converted before being aligned to the corresponding transformed genomes in a directional manner. After removing duplicated reads, the sequencing depth and coverage of methylcytosine were calculated. Methylated sites were identified using a binomial test based on the counts of methylated cytosines (mC), total counts (mC + nonmC), and the non-conversion rate (r). Sites with FDR-corrected *p*-values <0.05 were considered as methylated sites. The methylation level (ML) indicates the fraction of methylated Cs and was defined as ML (C) = reads (mC)/[reads (mC) + reads (nonmC)].

### 2.15 Annotation of genomic features and genes

Gene annotation data were obtained from the reference genome (*Sscrofa11.1*). The gene body region was defined as the region from the transcription start site (TSS) to the transcription termination site (TTS), and the promoter region was defined as the 2-kb region upstream of the TSS. The CpG dinucleotides tended to cluster in CpG contexts called CpG islands (CGIs), and CGIs were defined as regions >200 bp with a GC fraction >0.5 and an observed-to-expected ratio of CpG >0.65 on repeat-masked sequences as annotated by the UCSC Genome Browser. CGI shore was defined as the 2-kb regions extending in both directions from CGI. The UCSC Genome Browser was used to annotate the CGI and CGI shore (Haeussler et al., 2019).

### 2.16 Identification of DMRs and DMGs

DMRs were identified using the DSS software by estimating the dispersion parameter from gamma-Poisson or beta-binomial distributions (Park and Wu, 2016), and DMGs were defined as genes whose gene body region (from TSS to TTS) or promoter region (2 kb upstream from the TSS) overlapped with the DMRs.

### 2.17 Correlation analysis of DNA methylation and gene expression

To investigate the regulatory patterns of gene expression mediated by DNA methylation in Sertoli cells, genes were categorized into four groups (no expression, low expression, medium expression, and high expression) based on their expression levels using the quartile method. No expression, FPKM< 1; low expression, 1 ≤ FPKM < lower quartile; medium expression, lower quartile ≤ FPKM < upper quartile; high expression, upper quartile ≤ FPKM. We observed that the DNA methylation levels of the gene body and the 2 kb region extended in both directions from the gene body in the four gene groups. Spearman rank correlation analysis was performed to examine the relationship between the changes in DNA methylation levels and gene expression levels in DMGs.

### 2.18 Enrichment analysis

Gene ontology (GO) and Kyoto Encyclopedia of Genes and Genomes (KEGG) pathway enrichment analyses were performed using the OmicShare tools (https://www.omicshare.com/tools (accessed on 28 June 2023)). Both GO terms and KEGG pathways with a *p*-value <0.05 were considered nominally significant.

### 2.19 Statistical analysis

Each treatment group in the current study consisted of three independent biological replicates. A *t*-test was performed to compare the means between the experimental and control groups at a specific time point. The statistical analysis was conducted using SAS^®^ OnDemand for Academics (https://welcome.oda.sas.com/, (accessed on 1 July 2023)). Statistically significant differences were defined as *p* < 0.05.

## 3 Results

### 3.1 Expression of *DNMT3A* in Sertoli cells of juvenile testes

**FIGURE 1:**
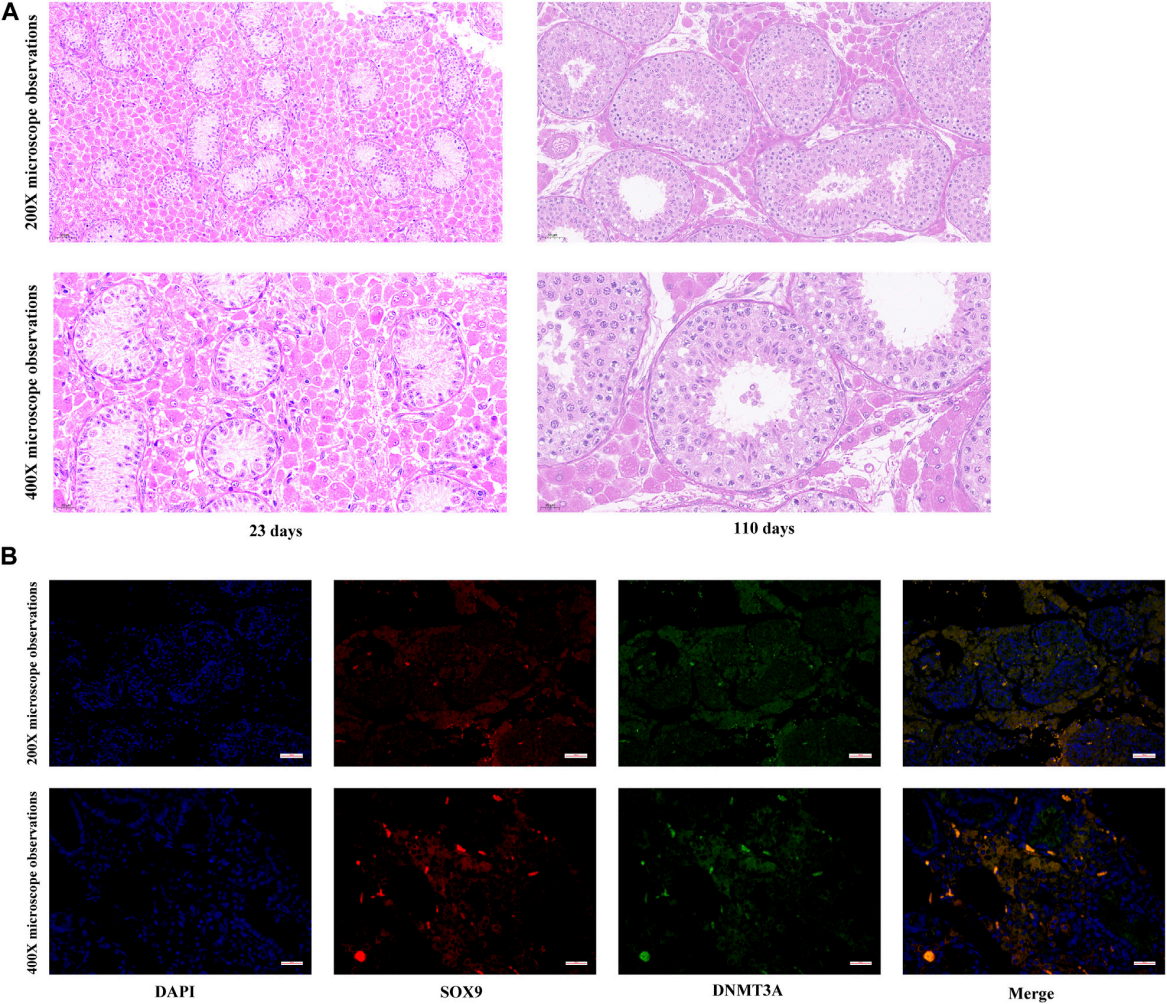
Expression of *DNMT3A* in Sertoli Cells. **(A)** Histological detection of testicular tissues from 23- and 110-days-old Shaziling pigs. **(B)** The location of DNMT3A in prepubertal porcine testicular tissue. SOX9 is marked in red, and DNMT3A is marked in green.

To elucidate developmental transformations in seminiferous tubules, Hematoxylin and Eosin staining was applied to porcine testes pre and post maturation. As depicted in Figure 1A, testicular maturation was accompanied by an increased number of cell layers and an expanded diameter within the seminiferous tubules. Moreover, a significant rise in the number of Sertoli cells in proximity to the basement membrane was observed. Notably, while our past research indicated a cessation of *DNMT3A* gene expression in Shaziling pig testes post-maturation, the absence of single-cell sequencing technology precluded definitive characterization of *DNMT3A* expression within pre-maturation Sertoli cells. To address this limitation, Immunofluorescence Staining was performed in the present study to unequivocally confirm *DNMT3A* expression in Sertoli cells prior to testicular maturation (Figure 1B).

### 3.2 *DNMT3A* inhibits the proliferation of immature Sertoli cells

**FIGURE 2:**
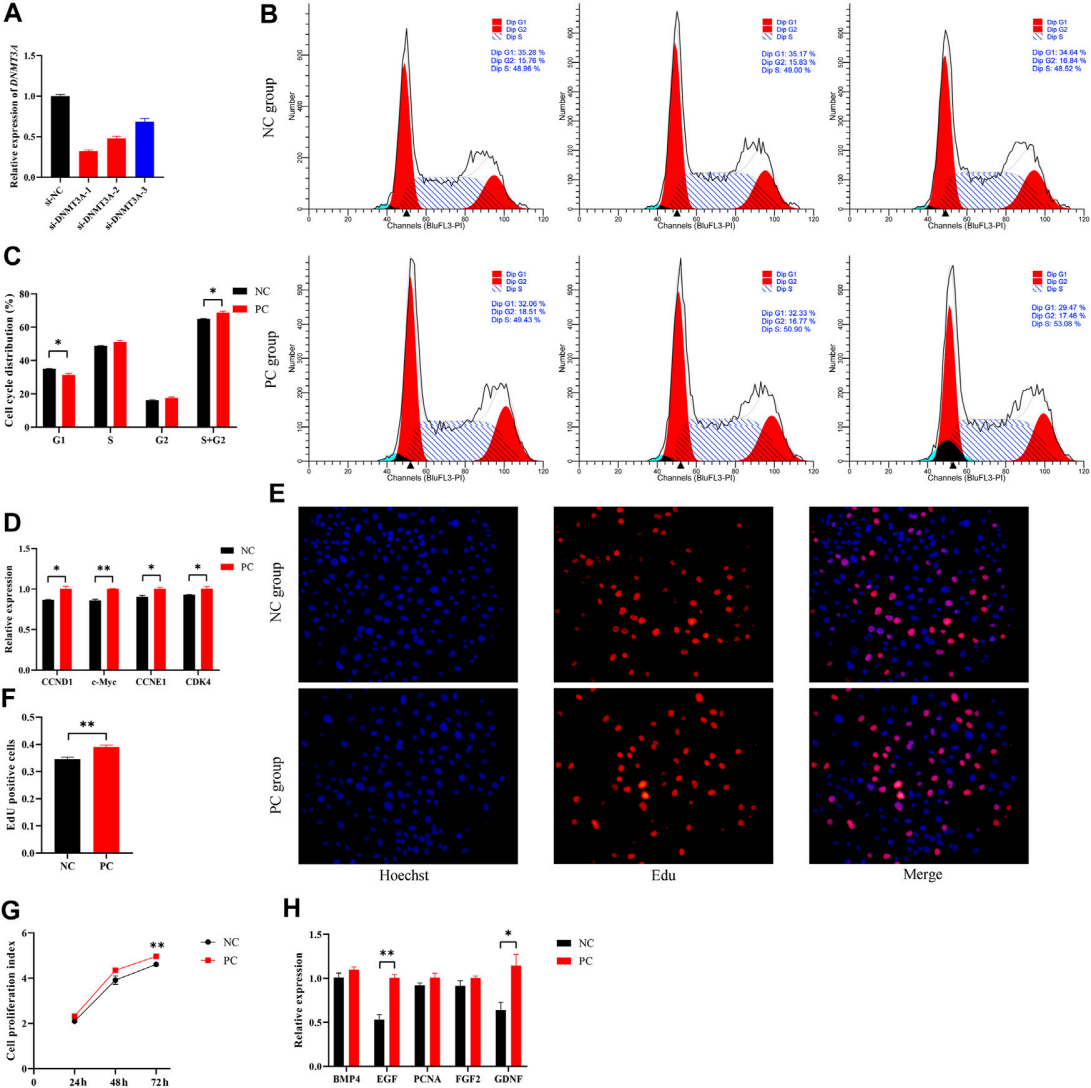
DNMT3A inhibits the proliferation of immature Sertoli cells. **(A)** Sertoli cells were treated with INHBA (si-DNMT3A-1, −2, −3). The inhibition efficacy was evaluated using the qPCR assay. si-NC, negative control of RNAi. **(B,C)** show the cell cycle was analyzed using a FACSCanto II flow cytometer, and the G1, S, and G2 phases of the cell cycle were counted in cells transfected with DNMT3A interference. **(D)** Relative mRNA expression of the key genes of cell proliferation. **(E)** Representative images of EdU staining of immature porcine Sertoli cells transfected with DNMT3A interference. **(F)** The proportion of EdU-positive cells in each treated cell group (*n* = 3). **(G)** The CCK-8 assay was used to measure the cell proliferation index. **(H)** Relative mRNA expression of the key genes of cell cycles. Data were presented as the mean ± SEM. *p < 0.05, **p < 0.01.

To simulate the expression pattern of *DNMT3A* during testicular development, the interference of *DNMT3A* was achieved using siRNA, and the siRNA with the highest interference efficiency was selected for further studies (Figure 2A). Additionally, Western blot experiments also demonstrated that the expression of DNMT3A protein was downregulated due to the interference (Supplementary Figure S1). To investigate the effect of *DNMT3A* on the immature Sertoli cells proliferation, cells were transfected with siRNA (referred to as the PC group) or si-NC (referred to as the NC group). The results of cell recycle assay indicated that interference with *DNMT3A* significantly reduced the proportion of immature Sertoli cells in the G1 phase (*p* < 0.05), while the total number of cells in the S phase + G2 phase significantly increased (*p* < 0.05) (Figures 2B,C). In addition, qPCR was performed to examine the expression levels of cell cycle-related genes (*CCND1*, *c-Myc*, *CCNE1*, and *CDK4*). As shown in Figure 2D, comparative analysis with the NC group revealed a significant upregulation in the expression levels of these genes upon *DNMT3A* downregulation (*p* < 0.05). These results provided evidence that *DNMT3A* suppressed the cell cycle progression of immature Sertoli cells.

Next, the 5-ethynyl-2′-deoxyuridine (EdU) incorporation assay showed that *DNMT3A* interference led to increased mitotic activity in Sertoli cells compared to the NC group (*p* < 0.05) (Figures 2E, F). Similarly, the Cell Counting Kit-8 (CCK-8) assay revealed that downregulation of *DNMT3A* expression significantly increased the proliferation index of Sertoli cells (*p* < 0.05) (Figure 2G). Additionally, *EGF* and *GDNF*, related to cell proliferation, were upregulated by *DNMT3A* interference (*p* < 0.05), while other cell proliferation-related genes, such as *BMP4*, *PCNA*, and *FGF*, exhibited a non-significant upward trend (*p* > 0.05) (Figure 2H). Taken together, these results revealed that *DNMT3A* inhibits immature porcine Sertoli cells proliferation.

### 3.3 *DNMT3A* promotes apoptosis of immature Sertoli cells

**FIGURE 3:**
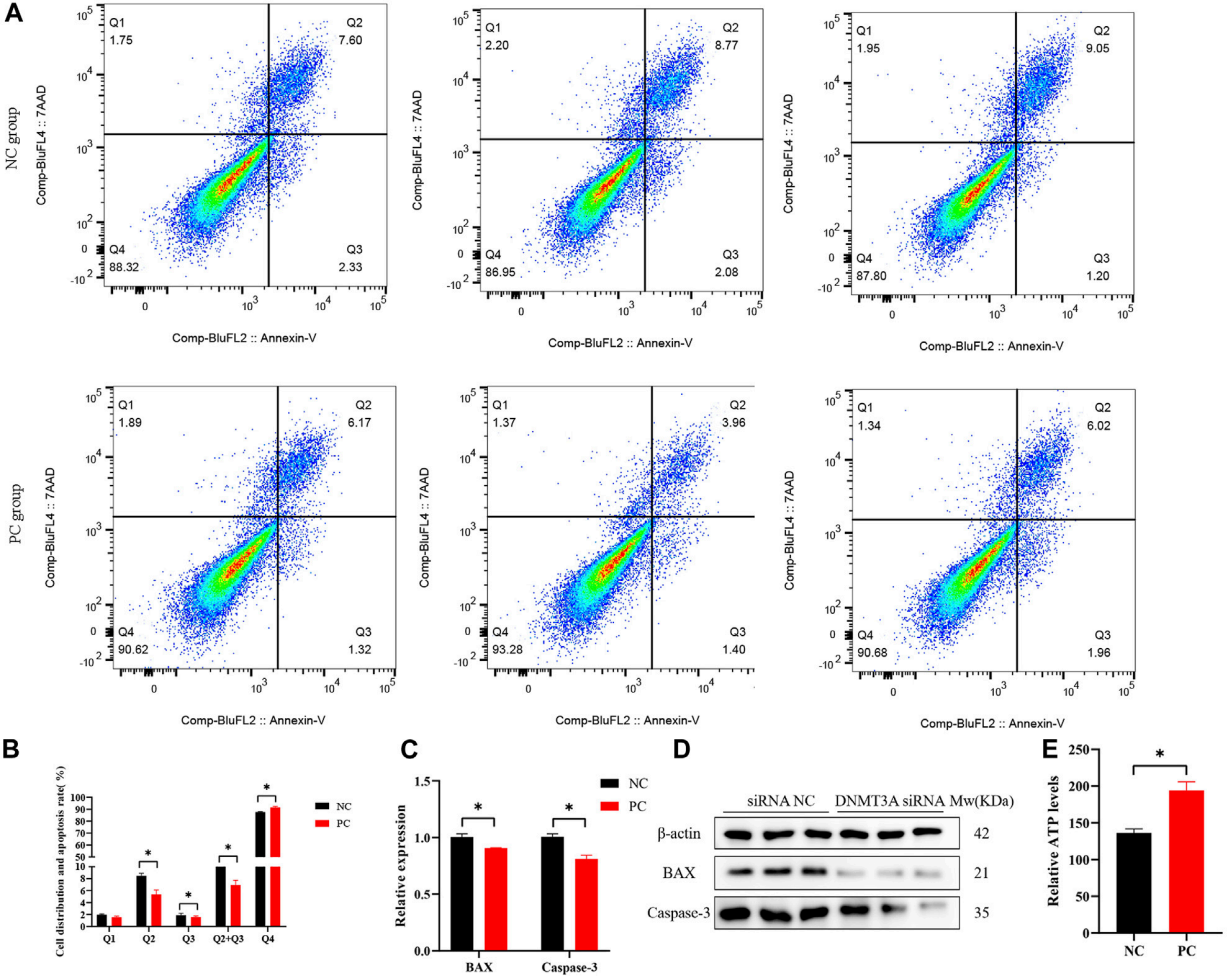
DNMT3A promotes apoptosis of Sertoli cells. **(A),(B)** Cell apoptosis phase distributions were detected using Annexin V-FITC/PI staining assay. Q1—the percentage of necrotic cells; Q2—the percentage of late apoptotic cells; Q3—the percentage of early apoptotic cells; Q4— non-apoptotic cells. **(C)** Relative mRNA expression of genes related to cell apoptosis. **(D)** Protein expression of cell apoptosis marker genes, and those proteins were determined using Western blot analysis. The β-actin gene was used as the internal control. **(E)** The relative ATP level was measured using an ATP assay kit. Data were presented as the mean ± SEM. *p < 0.05.

The regulatory roles underlying the effect of *DNMT3A* on apoptosis in Sertoli cells were also analyzed. The staining of annexin V and propidium iodide (PI) and flow cytometry analysis provided evidence that *DNMT3A* interference led to a significant decrease in the percentage of early and late apoptotic cells in Sertoli cells, consequently resulting in a significant reduction in the apoptotic rate (*p* < 0.05) (Figures 3A, B). The expression levels of apoptosis-related genes (BAX and Caspase-3) and proteins were significantly decreased (*p* < 0.05), as determined by qPCR and Western blot analysis (Figures 3C, D). The corresponding uncropped full-length blots were presented in Supplementary Figure S2. Furthermore, *DNMT3A* interference significantly increased intracellular ATP levels (Figure 3E) (*p* < 0.05), suggesting a potential association between *DNMT3A* and energy depletion, thereby influencing apoptosis in Sertoli cells. In summary, these results provide compelling evidence implicating DNMT3A in the promotion of apoptosis in immature Sertoli cells.

### 3.4 *DNMT3A* disruption alters the gene expression profile of immature Sertoli cells

**FIGURE 4:**
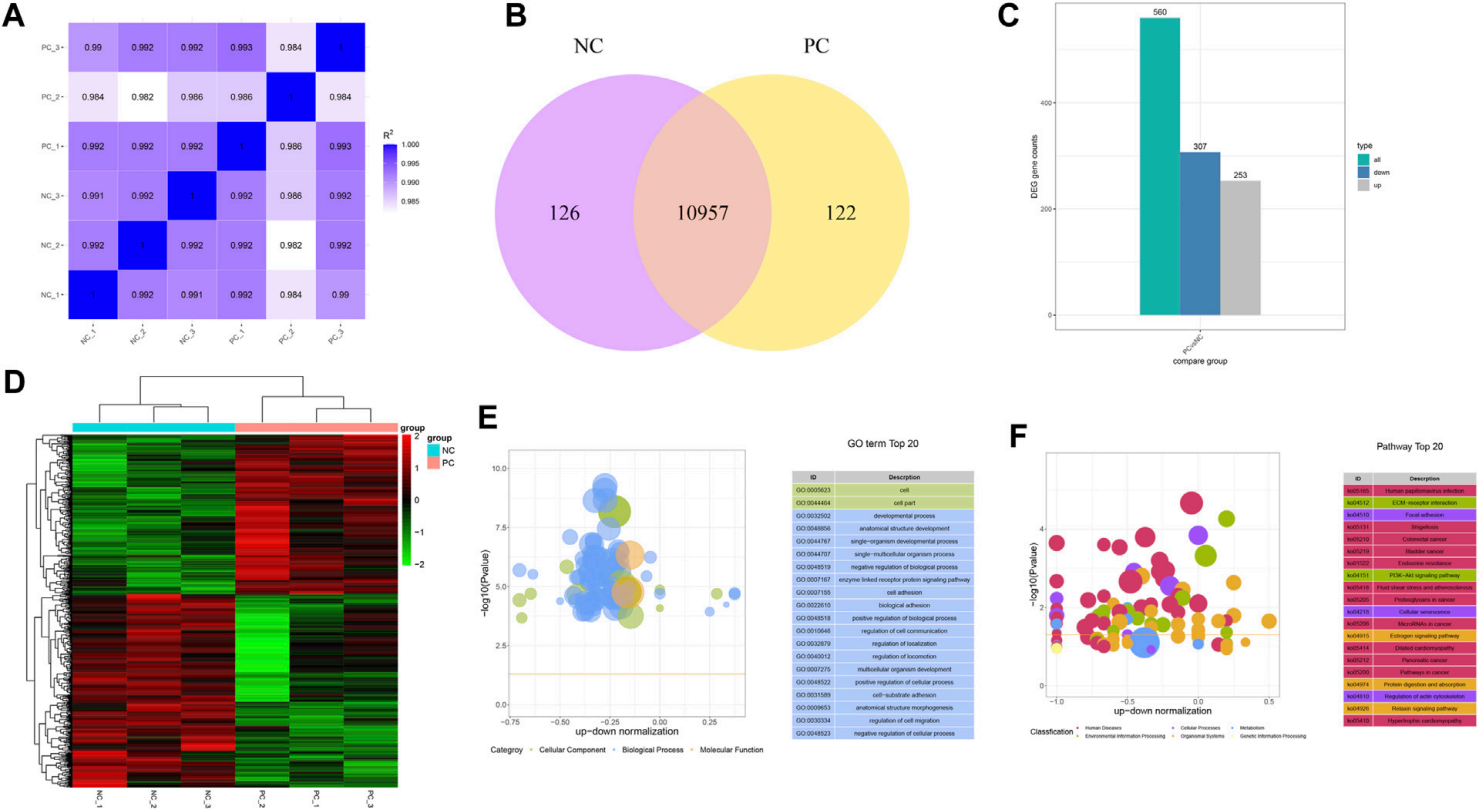
Genes expression patterns change by the DNMT interference. **(A)** Correlation analysis between each sample using FPKM. **(B)** Number of uniquely expressed genes in Sertoli cells under two treatment conditions. **(C)** Count of DEGs. **(D)** Cluster analysis for DEGs. Hierarchical clustering of gene FPKM values was performed, with normalization using Z-score. **(E)** Top 20 GO Terms Sorted by Significance. **(F)** The leading KEGG pathways.

To gain a deeper insight into the mechanism of how *DNMT3A* downregulation impacts the proliferation of immature Sertoli cells, RNA-seq was performed on variously treated immature Sertoli cells. A total of 43.23 Gb raw data was generated from the RNA-seq, and 272,379,880 clean reads covering 40.86 Gb of sequence of RNA-seq remained after data filtration. Bases with a Phred quality score (Q30) in the RNA-seq data from six samples accounted for 92.95%–93.86% of total, while unique alignment rates to the reference genome (*Sus scrofa 11.1*) spanned 92.09%–92.60%, indicating the high reliability of data for subsequent research (Supplementary Table S3). Following the quantification of the RNA-seq data from the six samples, the intergroup sample variation and intragroup sample reproducibility were depicted by calculating the Pearson correlation coefficient. As shown in Figure 4A, the high Pearson correlation coefficients for both intergroup variation and intragroup reproducibility suggested that *DNMT3A* interference significantly impacted the expression of only a subset of genes in the genome. For instance, 126 and 122 genes were exclusively expressed in the Sertoli cells of the NC group and PC group, respectively, and a total of 10,957 genes were found to be expressed in both groups (Figure 4B). Furthermore, a total of 560 differentially expressed genes (DEGs) were identified, of which 253 were upregulated and 307 were downregulated (Figure 4C), and these DEGs showed clear distinctions in their expression profiles between the two groups according to the cluster analysis (Figure 4D). Subsequently, the 560 DEGs were subjected to GO and KEGG enrichment analyses. The results of GO analysis showed significant associations between differentially expressed genes, influenced by *DNMT3A* interference, and key terms relevant to the development and function of immature Sertoli cells, such as developmental process (GO: 0032502), cell adhesion (GO: 0007167), and regulation of cell communication (GO: 0010646) (Figure 4E). Furthermore, the KEGG enrichment analysis demonstrated a strong correlation between the DEGs induced by *DNMT3A* and the development and maintenance of Sertoli cell function, including the Relaxin signaling pathway and the PI3K-Akt signaling pathway (Figure 4F). For example, previous studies have demonstrated that relaxin promotes the proliferation of Sertoli cells and enhances their population, thereby contributing to the maintenance of testicular tissue structure and function (Nascimento et al., 2012; Nascimento et al., 2016). Additionally, our previous study has demonstrated that the PI3K-Akt signaling pathway directly influences the proliferation of immature Sertoli cells (Luo et al., 2020). In conclusion, the results of RNA-Seq analysis indicated that downregulation of *DNMT3A* expression can influence immature testicular Sertoli cells through multiple mechanisms.

### 3.5 *DNMT3A* interference changes genomic DNA methylation patterns of immature Sertoli cells

RRBS was performed to enhance our understanding of the regulatory mechanisms by which *DNMT3A* influences gene expression through modulation of genomic methylation patterns in immature Sertoli cells. DNA methylation analysis of immature Sertoli cells was conducted using RRBS with >20× genome coverage and >98% conversion efficiency. A total of 70.41 Gb raw bases were generated, and after filtering out low-quality data, 56.43 Gb clean bases were obtained, with Q30 values ranging from 91.19% to 92.14% for the clean, full-length reads. The mapped reads, with mapping rates ranging from 69.66% to 72.43%, were utilized for subsequent analysis. Supplementary Table S4 provides detailed information on the sequencing data quality.

**FIGURE 5:**
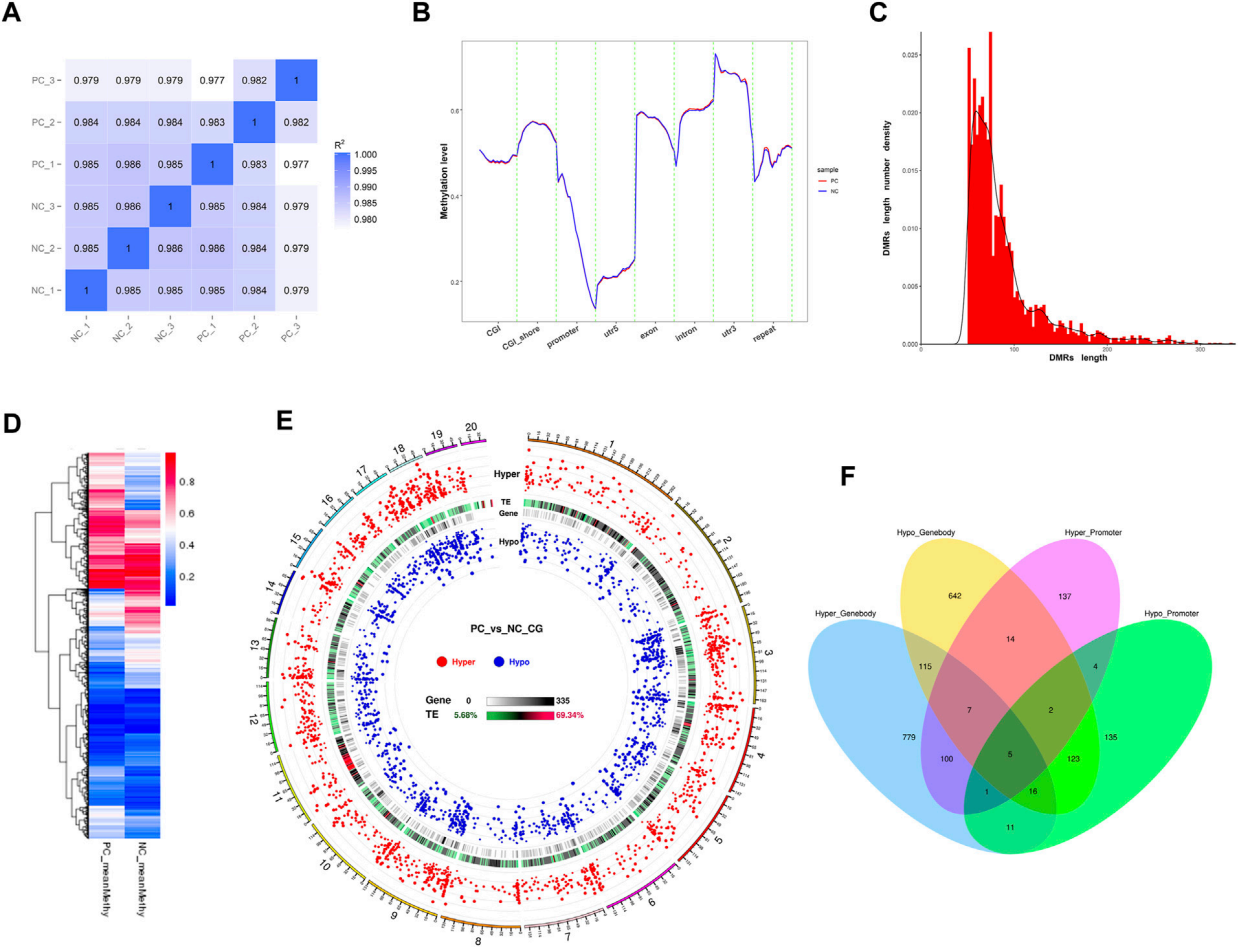
Genomic methylation patterns change by the DNMT interference. **(A)** Correlation analysis between each sample using common CpGs. **(B)** Distribution of methylation levels in genetic functional elements and feature sequences of two treated Sertoli cells. **(C)** The frequency distribution of DMR count by length. **(D)** Heatmap of methylation levels of DMRS between PC and NC groups. **(E)** Circos plots of the distribution and significance of two types of DMRs. The outer ring represents the chromosome of the reference genome, and the chromosome information corresponding to the number is listed in Supplementary Table S10. Circos plots were generated according to the length of the chromosome, from long to short: hyper—the distribution of the hypermethylated DMRs, in which the differential methylation level is positively correlated with the distance from the center of the circle; TE—the proportion of repetitive sequences, which turn from green to red, indicates the proportion from low to high; gene—the density of genes in each bin, in which turning from white to black indicates the density becoming larger; and hypo—the distribution of hypomethylated DMRs, in which the differential methylation level is negatively correlated with the distance from the center of the circle. **(F)** show the composition of DMGs associated with gene body and promoter. Hyper_Promoter—hypermethylated DMGs associated with promoters; Hypo_Promoter—hypomethylated DMGs associated with promoters; Hyper_Genebody—hypermethylated DMGs associated with gene bodies; and Hypo_Genebody—hypomethylated DMGs associated with gene bodies.

Pearson correlation analysis results demonstrated a high degree of repeatability in the sequencing data at the global CpG level of the methylation profiles (r > 0.982). However, the higher inter-group correlation coefficients suggested a relatively modest impact of *DNMT3A* interference on the genomic DNA methylation patterns of Sertoli cells (Figure 5A). Next, the methylation levels of various genomic feature sequences or functional elements were analyzed in the two groups of Sertoli cells. As shown in Figure 5B, *DNMT3A* interference significantly decreased the methylation levels of CGI, the 5′ untranslated region (5′UTR), exons, and promoter regions in Sertoli cells, while increasing the methylation levels of CGI shores and introns compared to the NC group. Furthermore, a total of 3,327 differentially methylated region (DMRs) were identified using the DSS software (Supplementary Table S5). The lengths of these DMRs ranged from 51 to 3,936 bp (Figure 5C), and their methylation levels exhibited significant differences between the two groups (Figure 5D). Notably, a considerable number of DMRs by *DNMT3A* interference had been identified in the genome, but only one DMR was found on the Y chromosome. Notably, numerous DMRs induced by *DNMT3A* interference were distributed across various genomic locations, whereas only one DMR was identified on the Y chromosome (Figure 5E).

Next, we identified differentially methylated genes (DMGs) when there was an overlap between the gene body region (from TSS to TTS) or the promoter region (2 kb upstream from the TSS) with DMRs. A total of 2,091 DMGs were detected between the two groups (Supplementary Table S6). Among them, 1,815 DMGs were identified in the gene body regions, including 1,034 hypermethylated DMGs and 924 hypomethylated DMGs. Additionally, 143 DMGs showed both hypermethylation and hypomethylation in the gene body regions. Furthermore, 555 DMGs were identified in the promoter regions, consisting of 270 hypermethylated DMGs and 297 hypomethylated DMGs. Among these, 12 DMGs exhibited both hypermethylation and hypomethylation in the promoter regions. Notably, 279 DMGs were shared between the promoter and gene body-associated DMRs (Figure 5F). Collectively, the downregulation of *DNMT3A* expression affects the genomic DNA methylation patterns in immature Sertoli cells.

### 3.6 The correlation between DNA methylation and gene expression

**FIGURE 6:**
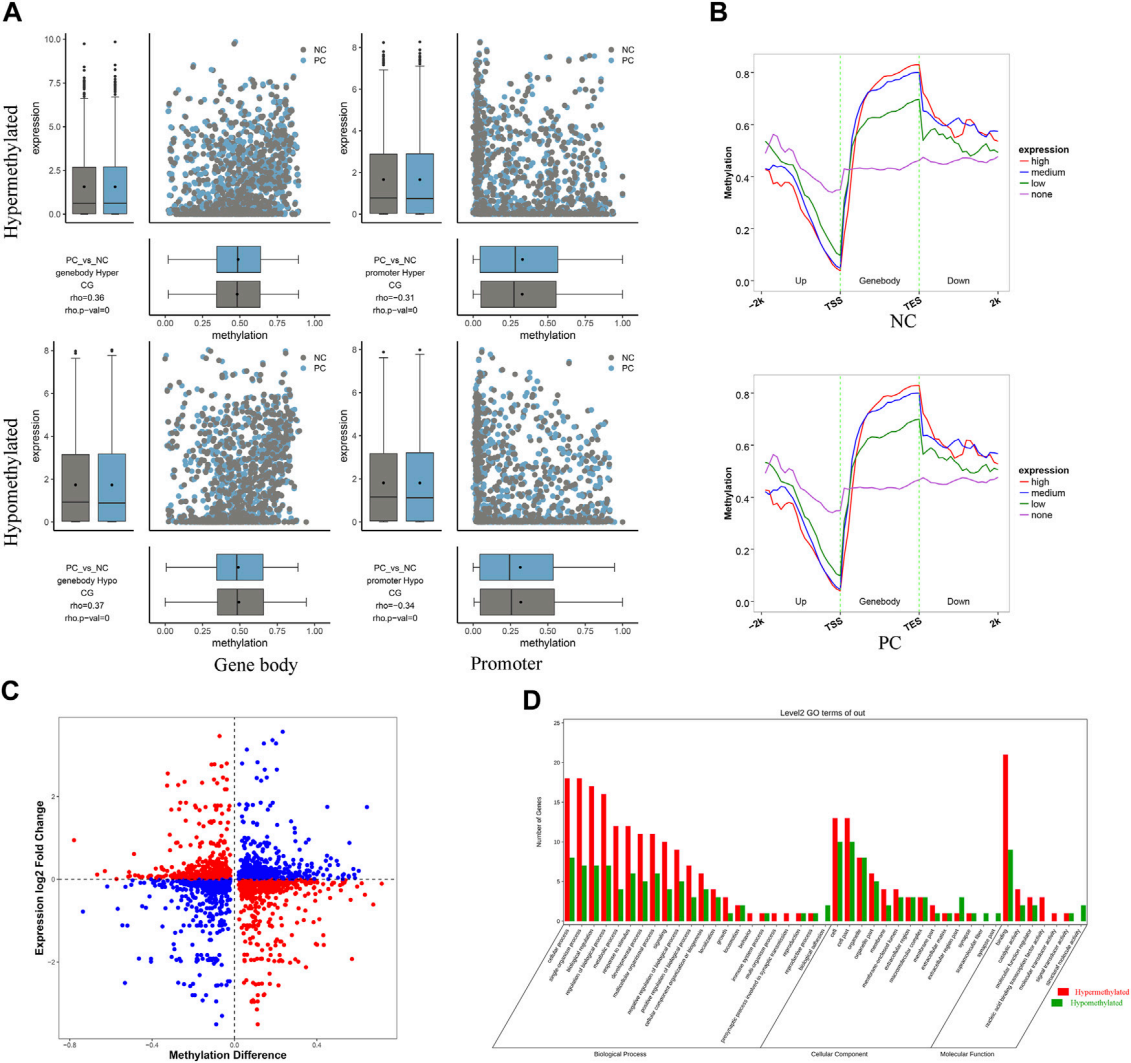
RRBS combined with RNA-seq analysis revealed the regulatory mechanisms of *DNMT3A* on Sertoli cell proliferation **(A)** Spearman rank correlation analysis was conducted to examine the relationship between methylation levels and corresponding expression levels of DMG associated with gene body and promoter. The horizontal axis represents the DMG methylation level, and the vertical axis represents the expression levels of DMGs **(B)** Average DNA methylation level in functional elements with different expression levels at each stage. Genes were classified into four groups according to their expression level (no expression, low expression, medium expression and high expression) by RNA-seq. **(C)** Differential methylation levels and corresponding differential expression levels of DMGs. **(D)** GO annotation was performed at Level 2 for three main categories.

The combined analysis of RRBS and RNA-seq results contributed to a comprehensive understanding of the intricate mechanisms underlying *DNMT3A*-mediated modification of the genomic DNA methylation patterns and its impact on Sertoli cell proliferation. Firstly, the corresponding relationship between the differential methylation levels and expression levels of DMGs of various types was demonstrated using a combination of scatter plots and box plots. As shown in Figure 6A, the Spearman rank correlation analysis revealed a negative correlation between the differential methylation levels and differential expression levels of DMGs associated with promoters (rho <0, *p* < 0.05), while a positive correlation was observed for DMGs associated with gene bodies (rho >0, *p* < 0.05). Then, to further elucidate the relationship between DNA methylation and gene expression in Sertoli cells, genes were categorized into four groups (no, low, medium, and high expression), and the corresponding DNA methylation levels in the 2k bp upstream and downstream regions of the gene body were presented. As depicted in Figure 6B, higher gene expression levels in the two groups of Sertoli cells corresponded to lower DNA methylation levels in the promoter regions, while higher gene expression levels were associated with higher DNA methylation levels in the gene body regions. Collectively, those results highlighted distinct negative and positive regulation between DNA methylation and gene expression in the promoter and gene body regions, respectively. However, it was worth noting that not all differential methylation levels of DMGs exhibited the expected relationship with differential expression levels as described above (Figure 6C), which can be explained by that the alteration of genomic methylation patterns by *DNMT3* interference is one of the several regulatory mechanisms for gene expression. For example, it has been reported that *DNMT3A* modulated chromatin accessibility through DNA methylation and thereby indirectly regulates gene expression (Wu and Zhang, 2014). Moreover, a total of 48 overlapping genes between DMGs associated with promoter and/or gene body and DEGs were collected for GO and KEGG enrichment analysis (Supplementary Table S7). The leading GO terms, annotated at Level 2 for three main categories (Biological process, cellular component, and molecular function), were presented in Figure 6D. For example, the leading terms of three categories were the septin ring (GO: 0005940), the vasculature development (GO: 0001944), and the protein binding (GO: 0005515) (Supplementary Table S8). Two significant pathways (Apelin signaling pathway and MicroRNAs in cancer) were identified by KEGG analysis (Supplementary Table S9). Collectively, those results provided evidences for the role of *DNMT3A* in altering genomic DNA methylation patterns and influencing immature proliferation.

## 4 Discussion

DNMT3A, a member of the DNA methyltransferase family, plays a critical role in establishing DNA methylation patterns during cell development and differentiation. In the context of Shaziling boar testicular development and maturation, DNMT3A displays differential expression as the sole member of the DNMT family (Anqi et al., 2022). Our previous research has demonstrated dynamic changes in genomic DNA methylation and gene expression patterns in the testes, emphasizing the regulatory role of DNA methylation in gene expression and its impact on testicular development (Anqi et al., 2022; Yang et al., 2023). However, considering the complexity of the testes as a reproductive organ comprising multiple cell types, our prior studies lacked cell-specific investigations, warranting further research. Thus, this study serves as a valuable complement to our previous research.

Several studies have provided evidence supporting *DNMT3A* as a positive regulator of cell proliferation. For example, Jing et al. (2019) and Qimuge et al. (2019) have demonstrated that *DNMT3A* promotes cell cycle progression and stimulates cell proliferation in specific cancer cell lines. These findings suggest that *DNMT3A* may enhance cell proliferation through its involvement in DNA methylation and gene regulation. Conversely, conflicting results have also been reported, suggesting a negative regulatory role of *DNMT3A* in cell proliferation. Studies by Challen et al. (Challen et al., 2011; Challen et al., 2014) have shown that conditional deletion of *DNMT3A* in hematopoietic stem cells (HSCs) resulted in a marked enhancement of self-renewal. The inconsistencies in the impact of *DNMT3A* on cell proliferation may be attributed to various factors, including cell type-specific effects, the cellular context, and variations in experimental conditions among studies. Furthermore, the expression level, activity, and interactions of *DNMT3A* with other regulatory factors may contribute to the observed discrepancies. Further investigations are required to unravel the precise mechanisms underlying the effects of *DNMT3A* on cell proliferation and to clarify the factors contributing to the reported inconsistencies. A comprehensive understanding of the complex and context-dependent role of *DNMT3A* in cell proliferation necessitates rigorous experimental approaches and considerations of multiple factors, which should be addressed in future studies to advance our knowledge in this field.

It is noteworthy that the number of DMGs identified produced by *DNMT3A* interference in this study was significantly greater than the number of DEGs, which can be explained by that DNA methylation is one of the several regulatory mechanisms governing gene expression (Hung and Chang, 2010; Frye et al., 2018). The integration of RRBS and RNA-seq data demonstrated that DNA methylation in promoters and gene bodies exerted a negative and positively regulatory effect on gene expression, respectively, which were consistent with our previous researches (Anqi et al., 2022; Yang et al., 2023). DNA methylation in the promoter region exerts a repressive effect on gene expression by inhibiting the binding of transcription factors and recruiting proteins with a high affinity for 5mC, leading to decreased activity of transcription activators (Sun et al., 2020; Kaluscha et al., 2022). However, the regulation of gene expression by gene body DNA methylation remains incompletely understood. It has been reported that gene-body methylation is highly conserved across eukaryotes, indicating that it has an important function (Feng et al., 2010; Zemach et al., 2010). To date, the positive regulatory mechanism of DNA methylation in gene bodies has not been fully elucidated, although two hypotheses have been proposed to explain this phenomenon: (1) 5 mC in a gene body facilitates co-transcriptional and/or splicing transcription elongation and (2) 5 mC in a gene body inhibits intragenic promoters (Greenberg and Bourchis, 2019).

KEGG enrichment analysis for overlapping genes between DMGs and DEGs revealed the regulatory mechanisms of *DNMT3A* on Sertoli cells proliferation. Apelin signaling pathway was identified as the leading pathway, which activated by the binding of apelin, a peptide hormone, to its specific receptor APJ (Apelin receptor) (Rak et al., 2017). Upon binding of apelin to the APJ receptor, the Apelin signaling pathway initiates a cascade of intracellular signaling events. One of the main downstream pathways is the G protein-dependent pathway, where activation of the APJ receptor leads to the activation of G proteins such as Gαq/11 and Gαi/o (Chen et al., 2020). This activation further stimulates intracellular effectors such as phospholipase C (PLC) (Lee et al., 2015), resulting in the generation of intracellular messengers like inositol trisphosphate (IP3) and diacylglycerol (DAG) (Alipour et al., 2017). IP3 triggers the release of calcium ions (Ca^2+^), while DAG activates protein kinase C (PKC), influencing cellular responses (Rasmussen et al., 1995). Another important pathway is the β-arrestin pathway, where upon receptor activation, β-arrestins are recruited to the activated APJ receptor (Zhang et al., 2023). This leads to receptor internalization and activation of intracellular signaling cascades, including MAPK pathways such as ERK and p38 MAPK (Wang et al., 2022). The Apelin signaling pathway can regulate gene expression (Trang et al., 2023), cell proliferation (Chaves-Almagro et al., 2022), and differentiation (Sisli et al., 2023), and is involved in the regulation of cardiovascular function, angiogenesis, fluid homeostasis, and energy metabolism (Respekta et al., 2022; Sisli et al., 2023). In the present study, genes significantly enriched in the Apelin signaling pathway included *APLN*, which encodes the Apelin protein. The expression of the *APLN* gene escalated in conjunction with an increase in methylation within the gene body region, following interference by *DNMT3A*, as shown in Supplementary Table S5. As suggested by Das et al. (2022), the enhancement of the Apelin/APJ signaling can significantly promote the proliferation and decrease apoptosis of testicular cells. In conjunction with our research findings, it can be inferred that *DNMT3A* influences the proliferation of Sertoli cells through the Apelin signaling pathway.

In summary, our study is the first to highlight the functional and regulatory roles of *DNMT3A* in the proliferation and apoptosis of the porcine Sertoli cells. The methylomic and transcriptomic landscapes generated from our study involving DNMT3A-interference-treated and si-NC-treated Sertoli cells provided valuable insights into testicular development. Additionally, they offered significant potential for furthering our understanding of male health, reproductive biology, and disease. Going forward, we planned to further explore other regulatory mechanisms of DNMT3A using ChIP-seq. Such investigations were expected to deepen our comprehensive understanding of the regulatory mechanisms underlying *DNMT3A*-mediated DNA methylation in normal testicular development.

## Data Availability

The original contributions presented in the study are publicly available. This data can be found here: https://ngdc.cncb.ac.cn/, accession numbers CRA011480 and CRA011482.

## Supplementary material

SUPPLEMENTARY FIGURE S1 Uncropped full-length blot of DNMT3A.

SUPPLEMENTARY FIGURE S2 Uncropped full-length blot.

SUPPLEMENTARY TABLE S1 Information on RNA interference for *DNMT3A.*

SUPPLEMENTARY TABLE S2 Primer information for qPCR used in the present study.

SUPPLEMENTARY TABLE S3 Sequencing statistics summary of the transcriptome data.

SUPPLEMENTARY TABLE S4 Sequencing statistics summary of the methylome data.

SUPPLEMENTARY TABLE S5 DMRs information. nCG represents the number of CpG sites. diff.Mehty, the differential levels of methylation. areaStat, the statistically significant, of which a higher absolute value indicates that the differences are more significant.

SUPPLEMENTARY TABLE S6 DMGs associated with the gene body and promoter.

SUPPLEMENTARY TABLE S7 Information of overlapping genes between DEGs and DMGs.

SUPPLEMENTARY TABLE S8 GO enrichment analysis results.

SUPPLEMENTARY TABLE S9 KEGG enrichment analysis results.

SUPPLEMENTARY TABLE S10 The chromosome information corresponding to the number.

## Abbreviations

DNMTs, DNA methytransferases; RRBS, reduced representation bisulfite sequencing; RNA-seq, RNA sequencing; EdU, 5-ethynyl-2′-deoxyuridine; PBS, phosphate-buffered saline; PI, propidium iodide; qPCR, quantitative Real-Time PCR; ATP, adenosine triphosphate; FPKM, fragments per kilobase of exon per million fragments; DEG, differentially expressed genes; 5mC, 5-methylcytosine; DMR, differentially methylated region; DMG, differentially methylated gene; TSS, transcription start site; TTS, transcription termination site; CpG or CG, cytosine and guanine dinucleotide; CGI, CpG island; CCK-8, Cell Counting Kit-8; RNAi, RNA interference; GO, Gene ontology; KEGG, Kyoto Encyclopedia of Genes and Genomes.

## References

[B1] Alipour F. G. Ashoori M. R. Pilehvar-Soltanahmadi Y. Zarghami N. (2017). An overview on biological functions and emerging therapeutic roles of apelin in diabetes mellitus. Diabetes Metab. Syndr. 11 (Suppl. 2), S919–S923. 10.1016/j.dsx.2017.07.016 28712823

[B2] Anders S. Huber W. (2010). Differential expression analysis for sequence count data. Genome Biol. 11 (10), R106. 10.1186/gb-2010-11-10-r106 20979621 PMC3218662

[B3] Anqi Y. Saina Y. Chujie C. Yanfei Y. Xiangwei T. Jiajia M. (2022). Regulation of DNA methylation during the testicular development of Shaziling pigs. Genomics 114 (5), 110450. 10.1016/j.ygeno.2022.110450 35995261

[B4] Challen G. A. Sun D. Jeong M. Luo M. Jelinek J. Berg J. S. (2011). Dnmt3a is essential for hematopoietic stem cell differentiation. Nat. Genet. 44 (1), 23–31. 10.1038/ng.1009 22138693 PMC3637952

[B5] Challen G. A. Sun D. Mayle A. Jeong M. Luo M. Rodriguez B. (2014). Dnmt3a and Dnmt3b have overlapping and distinct functions in hematopoietic stem cells. Cell Stem Cell 15 (3), 350–364. 10.1016/j.stem.2014.06.018 25130491 PMC4163922

[B6] Chaves-Almagro C. Auriau J. Dortignac A. Clerc P. Lulka H. Deleruyelle S. (2022). Upregulated apelin signaling in pancreatic cancer activates oncogenic signaling pathways to promote tumor development. Int. J. Mol. Sci. 23 (18), 10600. 10.3390/ijms231810600 36142542 PMC9503500

[B7] Chen J. Chen X. Li S. Jiang Y. Mao H. Zhang R. (2020). Individual phosphorylation sites at the C-terminus of the apelin receptor play different roles in signal transduction. Redox Biol. 36, 101629. 10.1016/j.redox.2020.101629 32863206 PMC7338617

[B8] Chen T. Chen X. Zhang S. Zhu J. Tang B. Wang A. (2021). The genome sequence archive family: toward explosive data growth and diverse data types. Genomics Proteomics Bioinforma. 19 (4), 578–583. 10.1016/j.gpb.2021.08.001 PMC9039563 34400360

[B9] Chen Z. Zhang Y. (2020). Role of mammalian DNA methyltransferases in development. Annu. Rev. Biochem. 89, 135–158. 10.1146/annurev-biochem-103019-102815 31815535

[B10] Dan J. Chen T. (2016). Genetic studies on mammalian DNA methyltransferases. Adv. Exp. Med. Biol. 945, 123–150. 10.1007/978-3-319-43624-1_6 27826837

[B11] Das M. Gurusubramanian G. Roy V. K. (2022). Postnatal developmental expression of apelin receptor proteins and its role in juvenile mice testis. J. Steroid Biochem. Mol. Biol. 224, 106178. 10.1016/j.jsbmb.2022.106178 36108814

[B12] Dura M. Teissandier A. Armand M. Barau J. Lapoujade C. Fouchet P. (2022). DNMT3A-dependent DNA methylation is required for spermatogonial stem cells to commit to spermatogenesis. Nat. Genet. 54 (4), 469–480. 10.1038/s41588-022-01040-z 35410378

[B13] Feng S. Cokus S. J. Zhang X. Chen P. Y. Bostick M. Goll M. G. (2010). Conservation and divergence of methylation patterning in plants and animals. Proc. Natl. Acad. Sci. U. S. A. 107 (19), 8689–8694. 10.1073/pnas.1002720107 20395551 PMC2889301

[B14] Frye M. Harada B. T. Behm M. He C. (2018). RNA modifications modulate gene expression during development. Science 361 (6409), 1346–1349. 10.1126/science.aau1646 30262497 PMC6436390

[B15] Greenberg M. V. C. Bourchis D. (2019). The diverse roles of DNA methylation in mammalian development and disease. Nat. Rev. Mol. Cell Biol. 20 (10), 590–607. 10.1038/s41580-019-0159-6 31399642

[B16] Griswold M. D. (2018). 50 years of spermatogenesis: Sertoli cells and their interactions with germ cells. Biol. Reprod. 99 (1), 87–100. 10.1093/biolre/ioy027 29462262 PMC7328471

[B17] Haeussler M. Zweig A. S. Tyner C. Speir M. L. Rosenbloom K. R. Raney B. J. (2019). The UCSC Genome Browser database: 2019 update. Nucleic Acids Res. 47 (D1), D853–D858. 10.1093/nar/gky1095 30407534 PMC6323953

[B18] Hung T. Chang H. Y. (2010). Long noncoding RNA in genome regulation: prospects and mechanisms. RNA Biol. 7 (5), 582–585. 10.4161/rna.7.5.13216 20930520 PMC3073254

[B19] Jing W. Song N. Liu Y. P. Qu X. J. Qi Y. F. Li C. (2019). DNMT3a promotes proliferation by activating the STAT3 signaling pathway and depressing apoptosis in pancreatic cancer. Cancer Manag. Res. 11, 6379–6396. 10.2147/CMAR.S201610 31372043 PMC6635825

[B20] Kaluscha S. Domcke S. Wirbelauer C. Stadler M. B. Durdu S. Burger L. (2022). Evidence that direct inhibition of transcription factor binding is the prevailing mode of gene and repeat repression by DNA methylation. Nat. Genet. 54 (12), 1895–1906. 10.1038/s41588-022-01241-6 36471082 PMC9729108

[B21] Kaneda M. Sado T. Hata K. Okano M. Tsujimoto N. Li E. (2004). Role of *de novo* DNA methyltransferases in initiation of genomic imprinting and X-chromosome inactivation. Cold Spring Harb. Symp. Quant. Biol. 69, 125–129. 10.1101/sqb.2004.69.125 16117641

[B22] Krueger F. Andrews S. R. (2011). Bismark: a flexible aligner and methylation caller for Bisulfite-Seq applications. Bioinformatics 27 (11), 1571–1572. 10.1093/bioinformatics/btr167 21493656 PMC3102221

[B23] Langmead B. Salzberg S. L. (2012). Fast gapped-read alignment with Bowtie 2. Nat. Methods 9 (4), 357–359. 10.1038/nmeth.1923 22388286 PMC3322381

[B24] Lee D. K. Jeong J. H. Oh S. Jo Y. H. (2015). Apelin-13 enhances arcuate POMC neuron activity via inhibiting M-current. PLoS One 10 (3), e0119457. 10.1371/journal.pone.0119457 25782002 PMC4363569

[B25] Liao Y. Smyth G. K. Shi W. (2014). featureCounts: an efficient general purpose program for assigning sequence reads to genomic features. Bioinformatics 30 (7), 923–930. 10.1093/bioinformatics/btt656 24227677

[B26] Luo H. Chen B. Weng B. Tang X. Chen Y. Yang A. (2020). miR-130a promotes immature porcine Sertoli cell growth by activating SMAD5 through the TGF-beta-PI3K/AKT signaling pathway. FASEB J. 34 (11), 15164–15179. 10.1096/fj.202001384R 32918760

[B27] Members C.-N. (2022). Partners. Database Resour. Natl. Genomics Data Cent. China Natl. Cent. Bioinformation 2022 Nucleic Acids Res 50 (D1), D27–D38. 10.1093/nar/gkab951 PMC8728233 34718731

[B28] Moore L. D. Le T. Fan G. (2013). DNA methylation and its basic function. Neuropsychopharmacology 38 (1), 23–38. 10.1038/npp.2012.112 22781841 PMC3521964

[B29] Mortazavi A. Williams B. A. McCue K. Schaeffer L. Wold B. (2008). Mapping and quantifying mammalian transcriptomes by RNA-Seq. Nat. Methods 5 (7), 621–628. 10.1038/nmeth.1226 18516045 PMC13303166

[B30] Nascimento A. R. Macheroni C. Lucas T. F. G. Porto C. S. Lazari M. F. M. (2016). Crosstalk between FSH and relaxin at the end of the proliferative stage of rat Sertoli cells. Reproduction 152 (6), 613–628. 10.1530/REP-16-0330 27601715

[B31] Nascimento A. R. Pimenta M. T. Lucas T. F. G. Royer C. Porto C. S. Lazari M. F. M. (2012). Intracellular signaling pathways involved in the relaxin-induced proliferation of rat Sertoli cells. Eur. J. Pharmacol. 691 (1-3), 283–291. 10.1016/j.ejphar.2012.07.021 22819701

[B32] Ni F. D. Hao S. L. Yang W. X. (2019). Multiple signaling pathways in Sertoli cells: recent findings in spermatogenesis. Cell Death Dis. 10 (8), 541. 10.1038/s41419-019-1782-z 31316051 PMC6637205

[B33] Orth J. M. Gunsalus G. L. Lamperti A. A. (1988). Evidence from Sertoli cell-depleted rats indicates that spermatid number in adults depends on numbers of Sertoli cells produced during perinatal development. Endocrinology 122 (3), 787–794. 10.1210/endo-122-3-787 3125042

[B34] Park Y. Wu H. (2016). Differential methylation analysis for BS-seq data under general experimental design. Bioinformatics 32 (10), 1446–1453. 10.1093/bioinformatics/btw026 26819470 PMC12157722

[B35] Pertea M. Kim D. Pertea G. M. Leek J. T. Salzberg S. L. (2016). Transcript-level expression analysis of RNA-seq experiments with HISAT, StringTie and Ballgown. Nat. Protoc. 11 (9), 1650–1667. 10.1038/nprot.2016.095 27560171 PMC5032908

[B36] Qimuge N. He Z. Qin J. Sun Y. Wang X. Yu T. (2019). Overexpression of DNMT3A promotes proliferation and inhibits differentiation of porcine intramuscular preadipocytes by methylating p21 and PPARg promoters. Gene 696, 54–62. 10.1016/j.gene.2019.02.029 30772521

[B37] Rak A. Drwal E. Rame C. Knapczyk-Stwora K. Słomczyńska M. Dupont J. (2017). Expression of apelin and apelin receptor (APJ) in porcine ovarian follicles and *in vitro* effect of apelin on steroidogenesis and proliferation through APJ activation and different signaling pathways. Theriogenology 96, 126–135. 10.1016/j.theriogenology.2017.04.014 28532828

[B38] Rasmussen H. Isales C. M. Calle R. Throckmorton D. Anderson M. Gasalla-Herraiz J. (1995). Diacylglycerol production, Ca2+ influx, and protein kinase C activation in sustained cellular responses. Endocr. Rev. 16 (5), 649–681. 10.1210/edrv-16-5-649 8529575

[B39] Respekta N. Pich K. Dawid M. Mlyczyńska E. Kurowska P. Rak A. (2022). The apelinergic system: apelin, ELABELA, and APJ action on cell apoptosis: anti-apoptotic or pro-apoptotic effect? Cells 12 (1), 150. 10.3390/cells12010150 36611944 PMC9818302

[B40] Sisli H. B. Şenkal S. Hayal T. B. Bulut E. Doğan A. (2023). Regulatory role of apelin receptor signaling in migration and differentiation of mouse embryonic stem cell-derived mesoderm cells and mesenchymal stem/stromal cells. Hum. Cell 36 (2), 612–630. 10.1007/s13577-023-00861-2 36692671

[B41] Sun X. Xiao L. Chen J. Chen X. Chen X. Yao S. (2020). DNA methylation is involved in the pathogenesis of osteoarthritis by regulating CtBP expression and CtBP-mediated signaling. Int. J. Biol. Sci. 16 (6), 994–1009. 10.7150/ijbs.39945 32140068 PMC7053340

[B42] Trang N. T. N. Lai C. Y. Tsai H. C. Huang Y. L. Liu S. C. Tsai C. H. (2023). Apelin promotes osteosarcoma metastasis by upregulating PLOD2 expression via the Hippo signaling pathway and hsa_circ_0000004/miR-1303 axis. Int. J. Biol. Sci. 19 (2), 412–425. 10.7150/ijbs.77688 36632453 PMC9830518

[B43] Wang X. Zhang L. Zheng Y. Yang Y. Ji S. (2022). Apelin/APJ system in inflammation. Int. Immunopharmacol. 109, 108822. 10.1016/j.intimp.2022.108822 35605524

[B44] Wong W. J. Khan Y. S. (2022). “Histology, Sertoli cell,” in StatPearls (Treasure Island (FL). 32809466

[B45] Wu H. Zhang Y. (2014). Reversing DNA methylation: mechanisms, genomics, and biological functions. Cell 156 (1-2), 45–68. 10.1016/j.cell.2013.12.019 24439369 PMC3938284

[B46] Yang A. Yan S. Yin Y. Chen C. Tang X. Ran M. (2023). FZD7, regulated by non-CpG methylation, plays an important role in immature porcine Sertoli cell proliferation. Int. J. Mol. Sci. 24 (7), 6179. 10.3390/ijms24076179 37047150 PMC10094452

[B47] Young M. D. Wakefield M. J. Smyth G. K. Oshlack A. (2010). Gene ontology analysis for RNA-seq: accounting for selection bias. Genome Biol. 11 (2), R14. 10.1186/gb-2010-11-2-r14 20132535 PMC2872874

[B48] Zemach A. McDaniel I. E. Silva P. Zilberman D. (2010). Genome-wide evolutionary analysis of eukaryotic DNA methylation. Science 328 (5980), 916–919. 10.1126/science.1186366 20395474

[B49] Zhang H. Chen J. Shi M. Xu F. Zhang X. Gong D. W. (2023). Comparative study of elabela and apelin on apelin receptor activation through beta-arrestin recruitment. Mol. Biotechnol. 65 (3), 394–400. 10.1007/s12033-022-00529-6 35960440 PMC9935735

[B50] Zhang Z. Cao Y. Zhai Y. Ma X. An X. Zhang S. (2018). MicroRNA-29b regulates DNA methylation by targeting Dnmt3a/3b and Tet1/2/3 in porcine early embryo development. Dev. Growth Differ. 60 (4), 197–204. 10.1111/dgd.12537 29878317

[B51] Zhao L. Yao C. Xing X. Jing T. Li P. Zhu Z. (2020). Single-cell analysis of developing and azoospermia human testicles reveals central role of Sertoli cells. Nat. Commun. 11 (1), 5683. 10.1038/s41467-020-19414-4 33173058 PMC7655944

