# Supplementary figures and images for: Integration of RRBS and RNA-seq unravels the regulatory role of DNMT3A in porcine Sertoli cell proliferation
Source: Front Genet. 2024 Jan 9;14:1302351. doi: 10.3389/fgene.2023.1302351 (PMC10803568; doi:10.3389/fgene.2023.1302351)

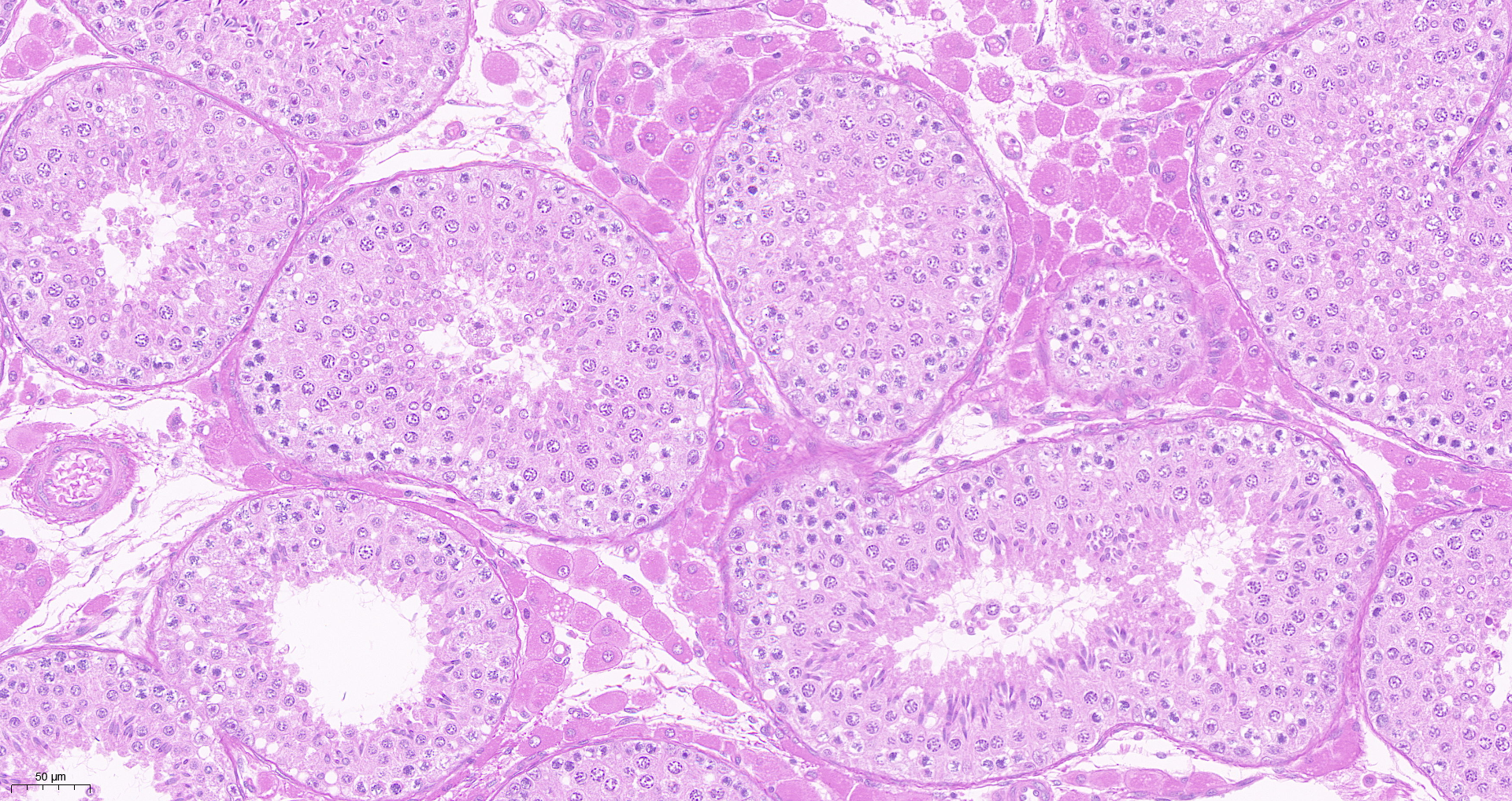

Supplement: Supplementary file 2 [file DataSheet3.ZIP › HE/110d B-1 TXW5.26-2_20.0x.tif]

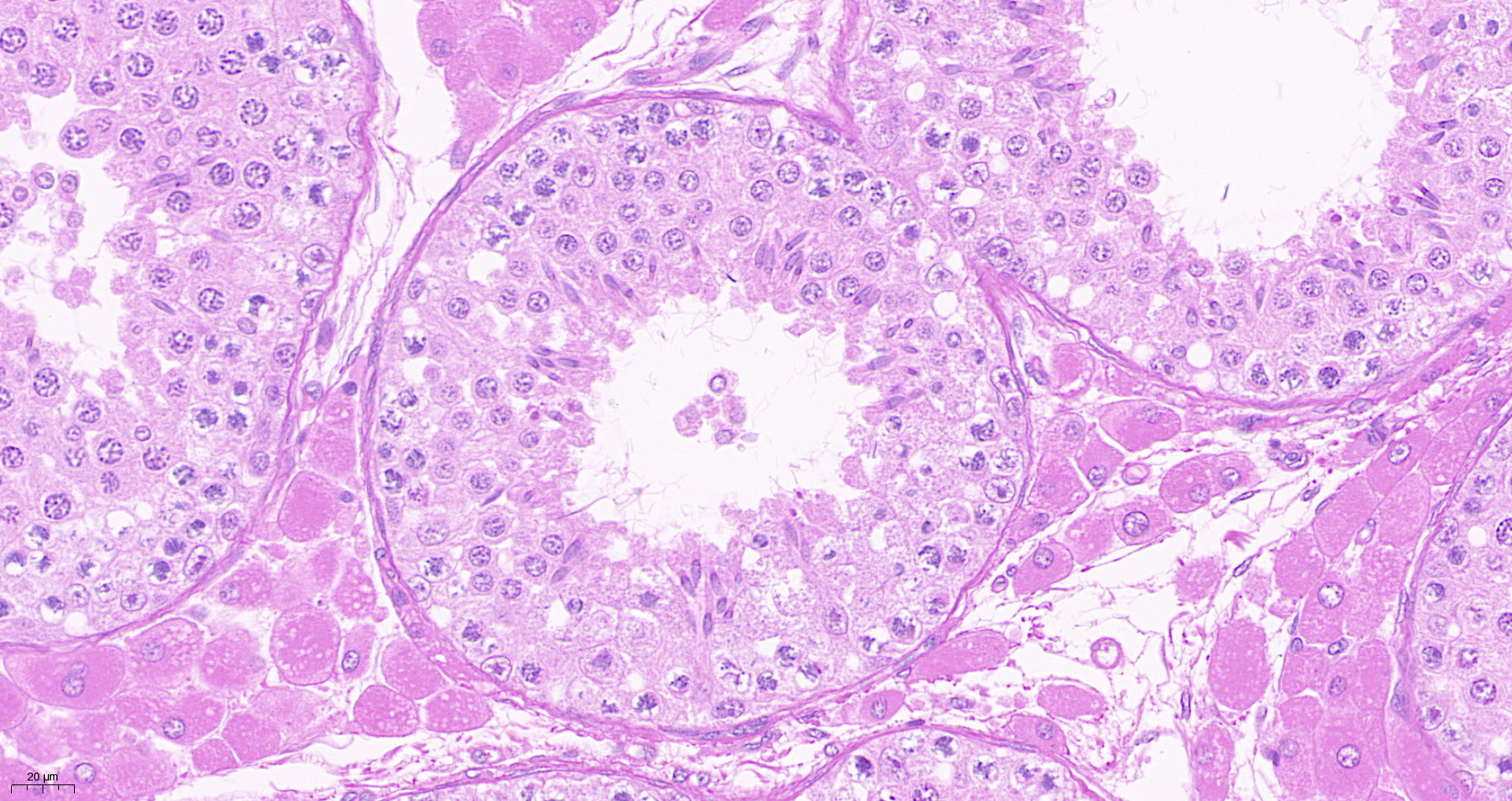

Supplement: Supplementary file 2 [file DataSheet3.ZIP › HE/110d B-1 TXW5.26-2_40.0x.tif]

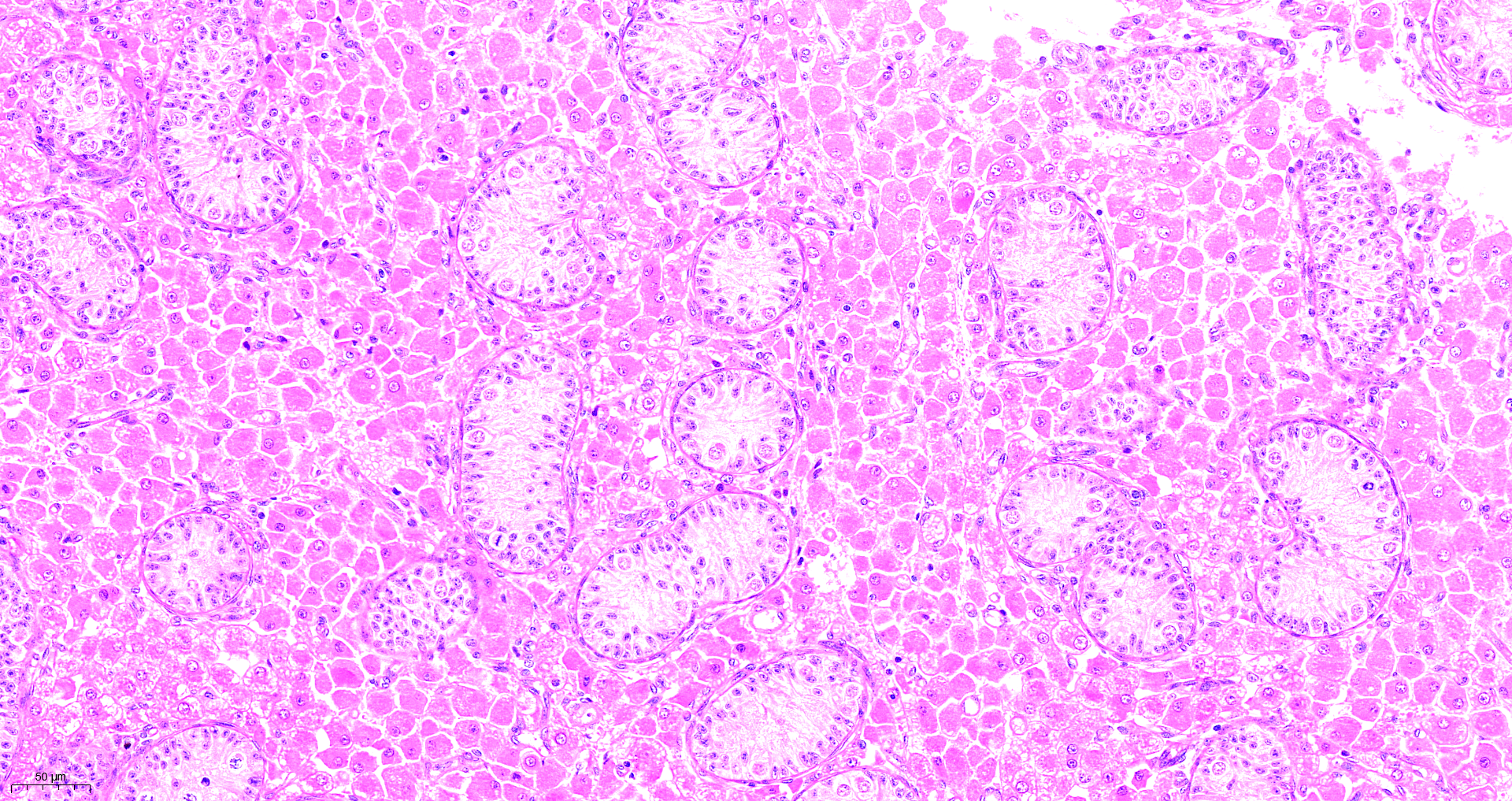

Supplement: Supplementary file 2 [file DataSheet3.ZIP › HE/23d GW 22.4.18 TXW-11 TXW+22.9.2_20.0x.tif]

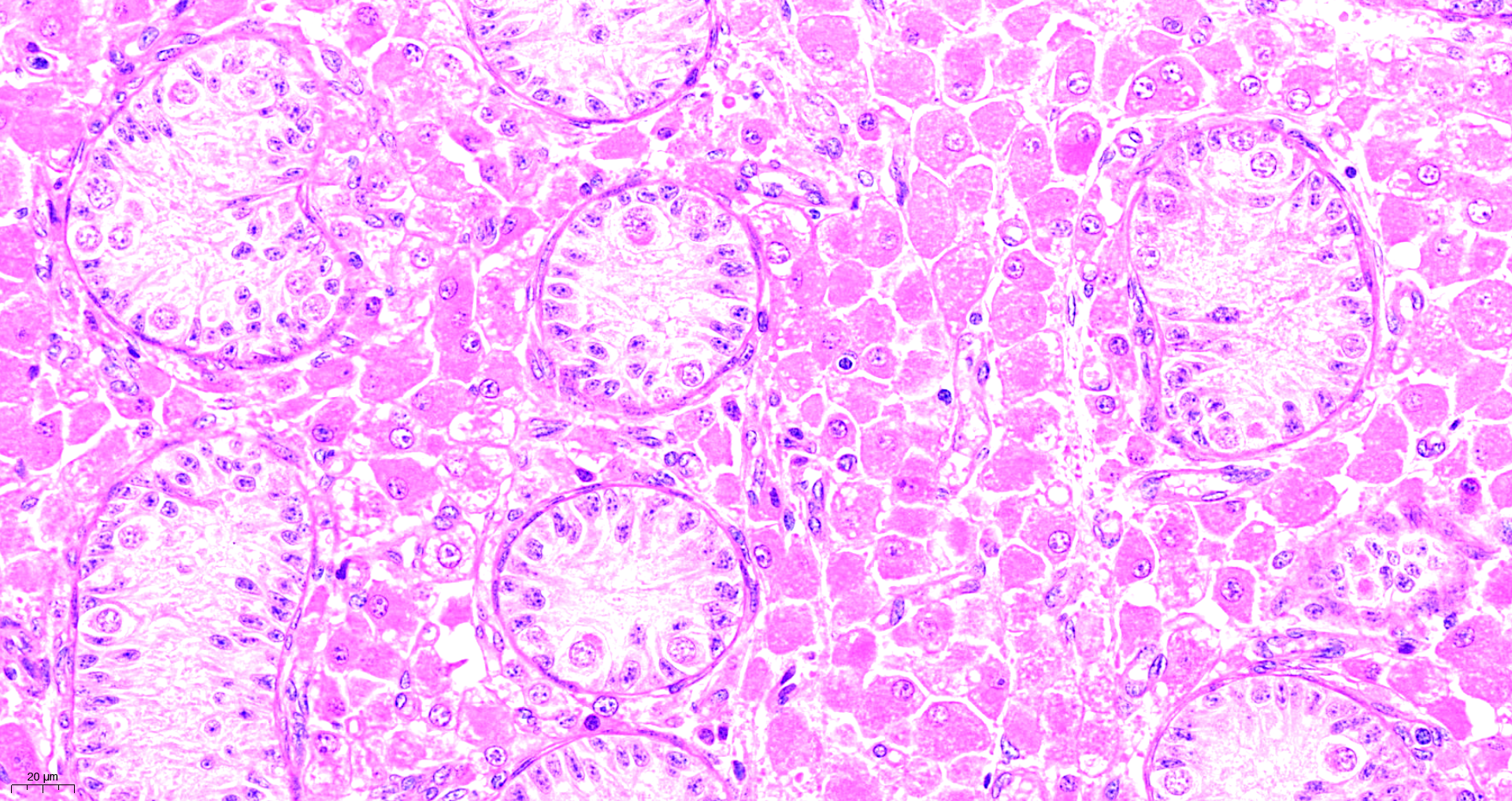

Supplement: Supplementary file 2 [file DataSheet3.ZIP › HE/23d GW 22.4.18 TXW-11 TXW+22.9.2_40.0x.tif]

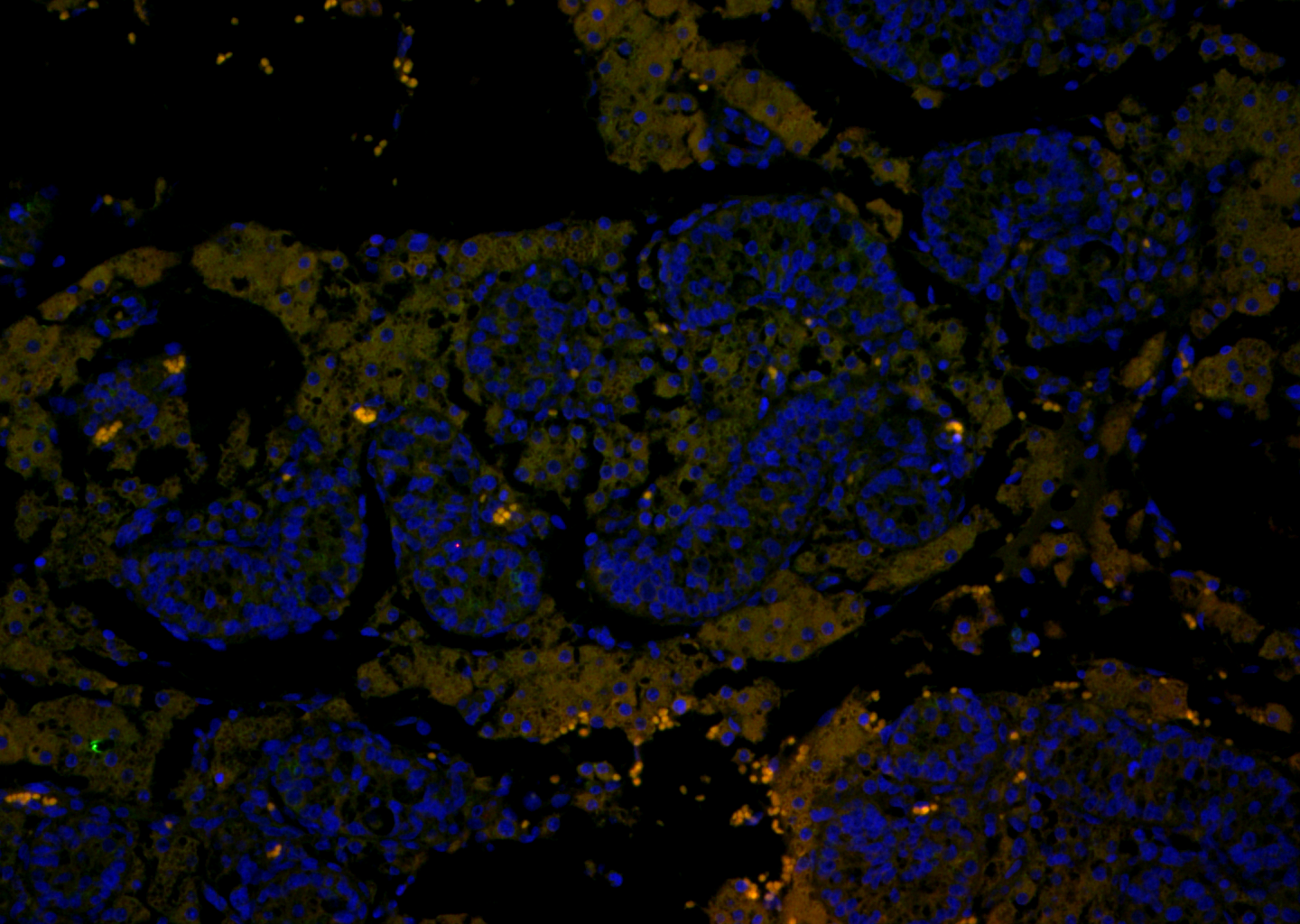

Supplement: Supplementary file 7 [file DataSheet4.ZIP › Immunofluorescence/6日睾丸原位杂交固定(大) SOX9红+DNMT39绿 200-1+2+3.tif]

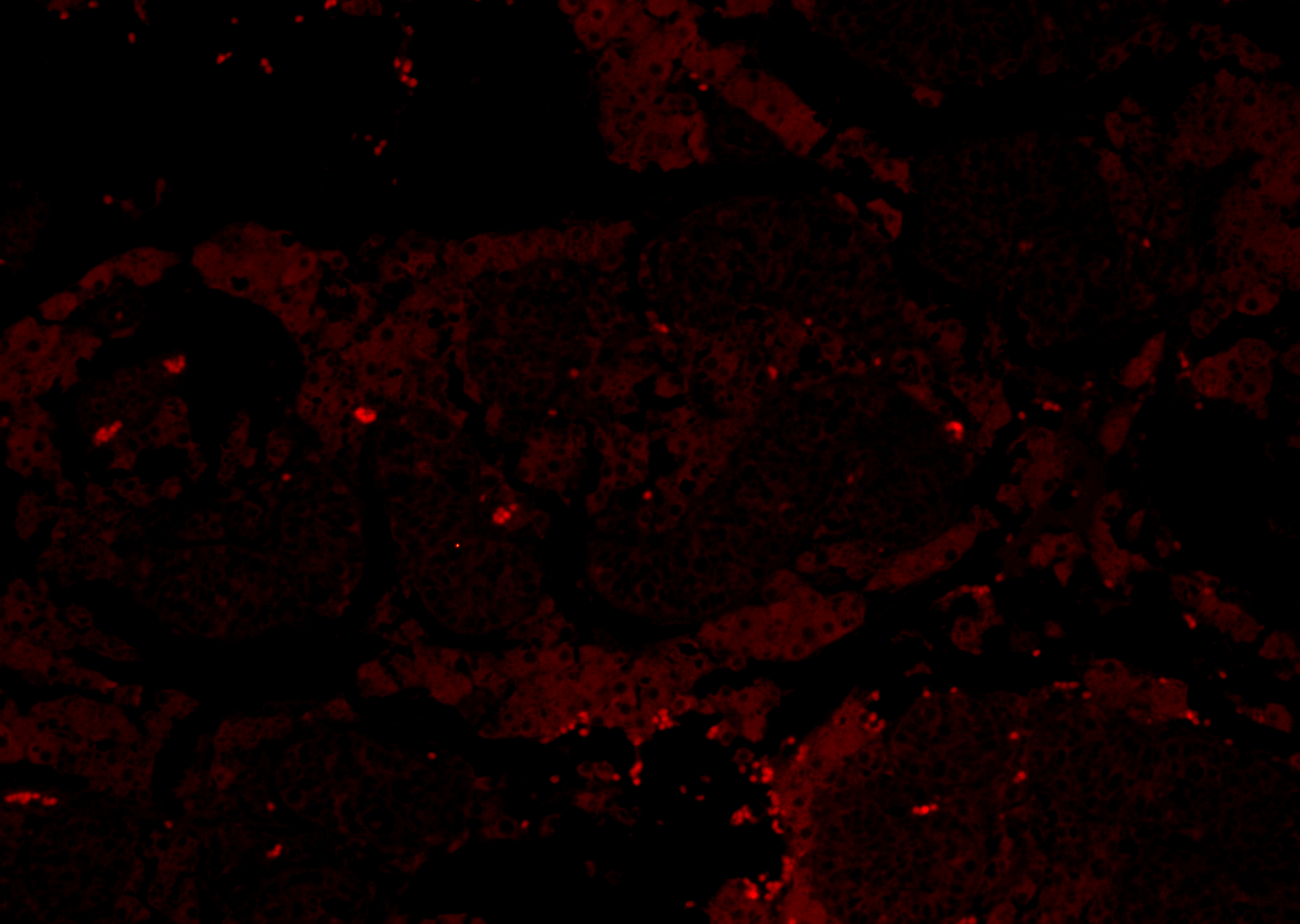

Supplement: Supplementary file 7 [file DataSheet4.ZIP › Immunofluorescence/6日睾丸原位杂交固定(大) SOX9红+DNMT39绿 200-1.tif]

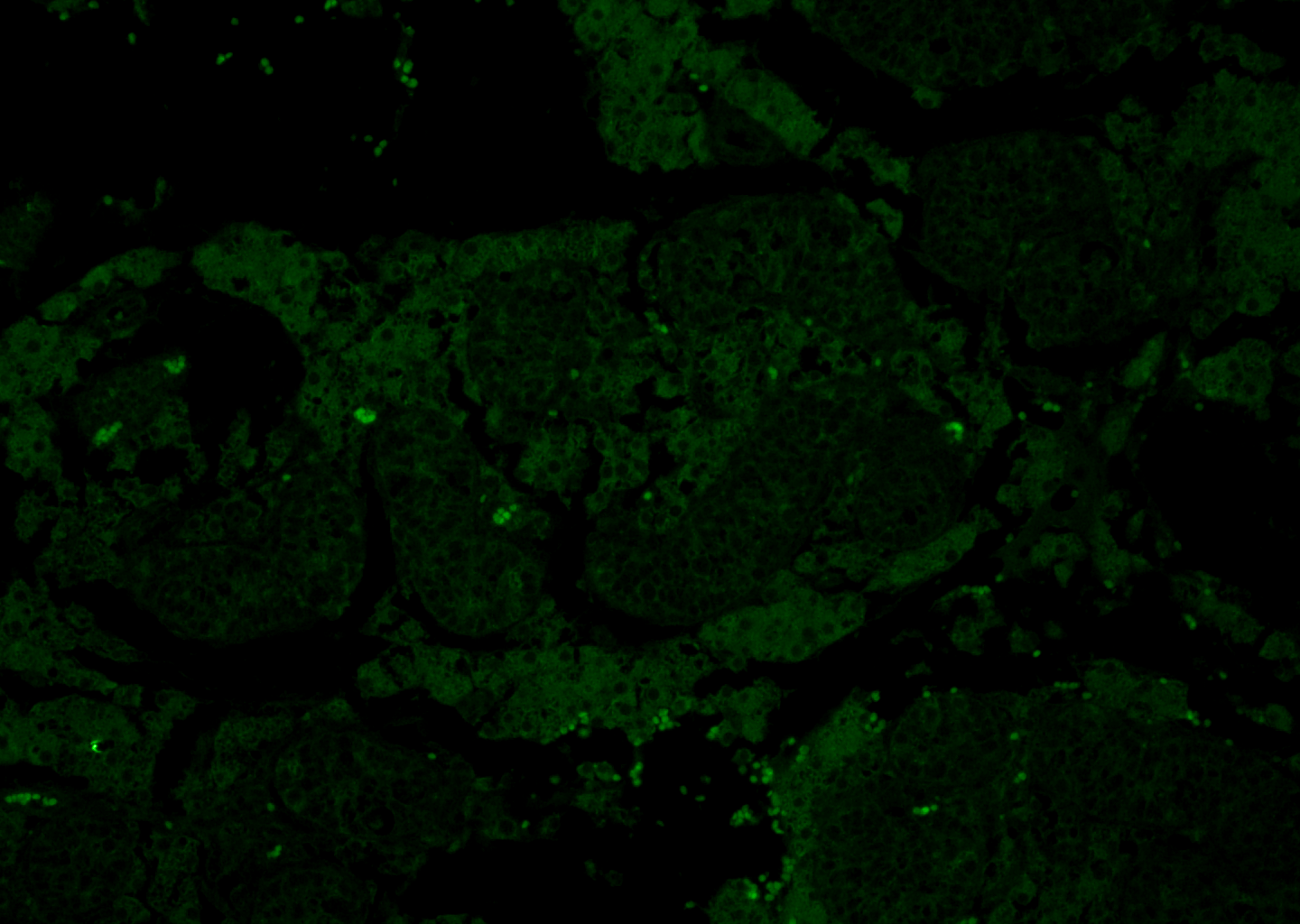

Supplement: Supplementary file 7 [file DataSheet4.ZIP › Immunofluorescence/6日睾丸原位杂交固定(大) SOX9红+DNMT39绿 200-2.tif]

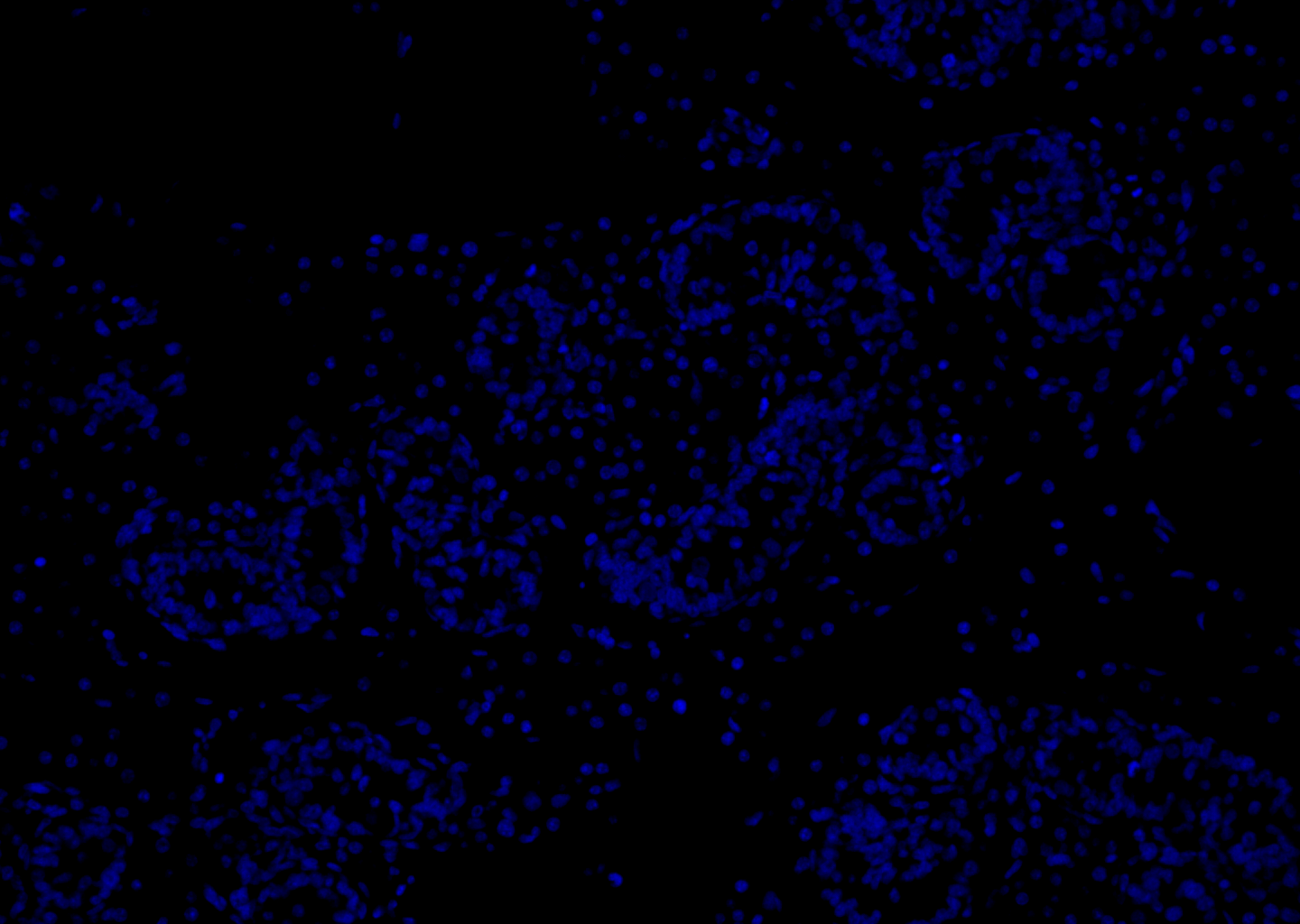

Supplement: Supplementary file 7 [file DataSheet4.ZIP › Immunofluorescence/6日睾丸原位杂交固定(大) SOX9红+DNMT39绿 200-3.tif]

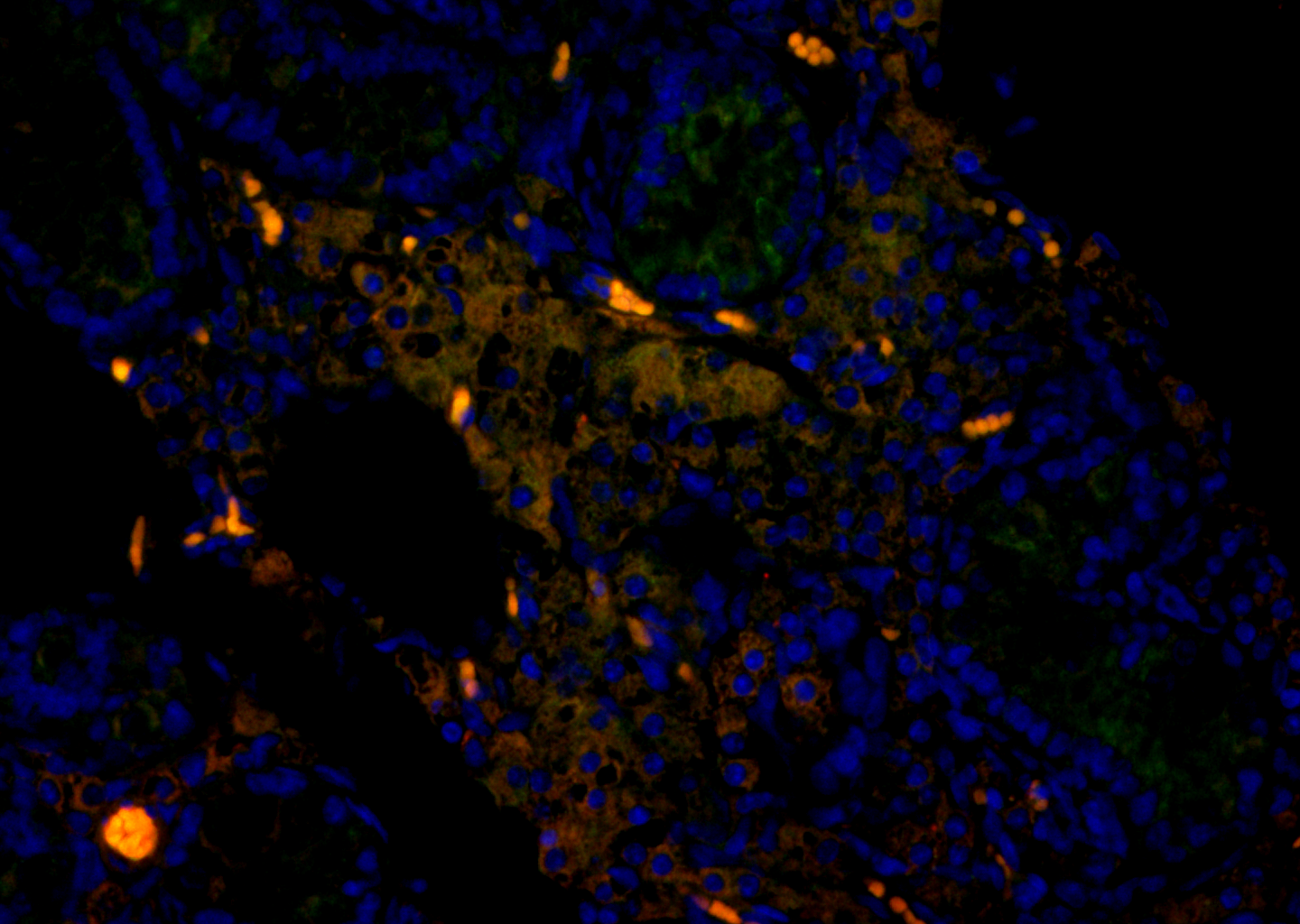

Supplement: Supplementary file 7 [file DataSheet4.ZIP › Immunofluorescence/6日睾丸原位杂交固定(小) SOX9红+DNMT39绿 400-7+8+9.tif]

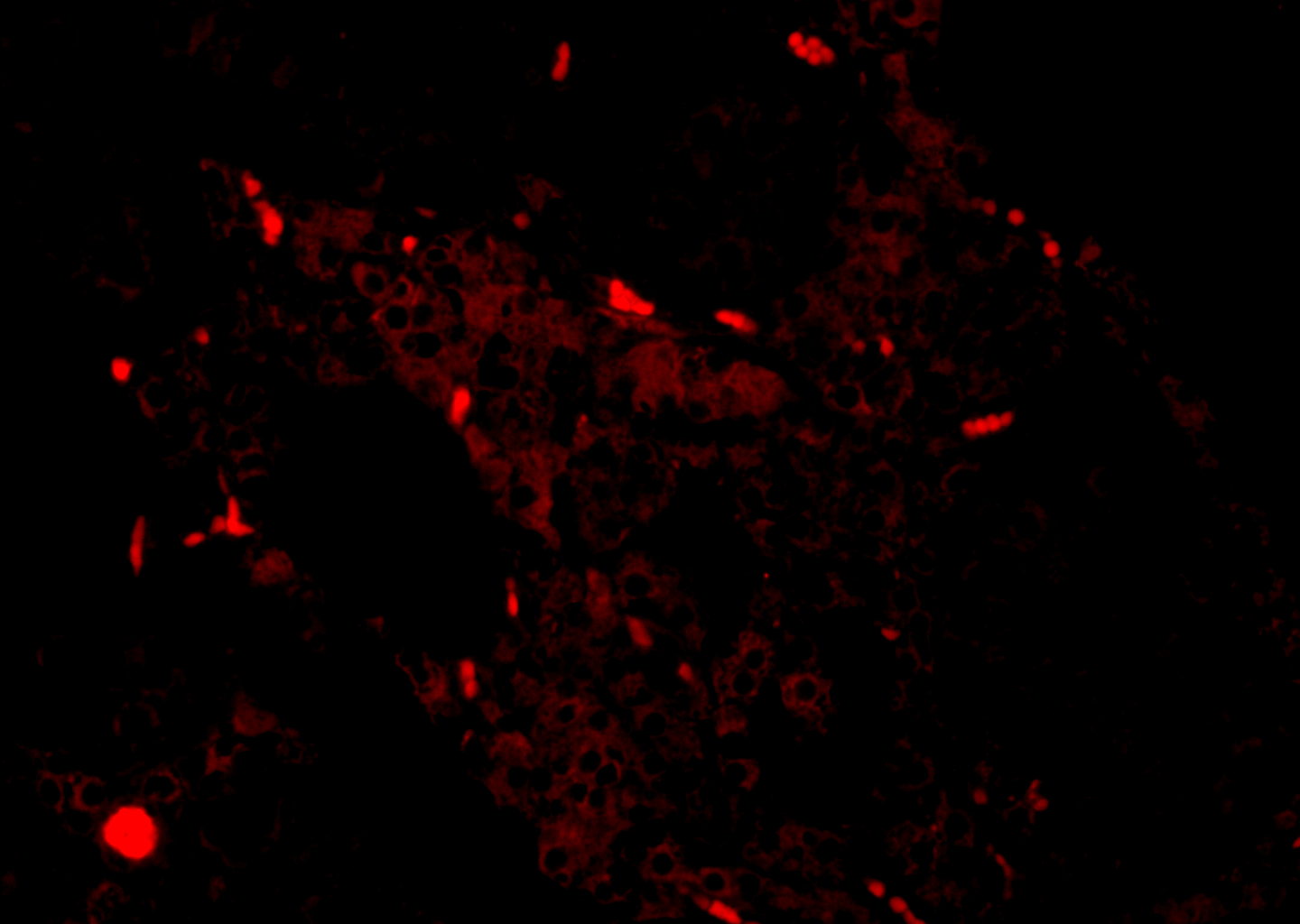

Supplement: Supplementary file 7 [file DataSheet4.ZIP › Immunofluorescence/6日睾丸原位杂交固定(小) SOX9红+DNMT39绿 400-7.tif]

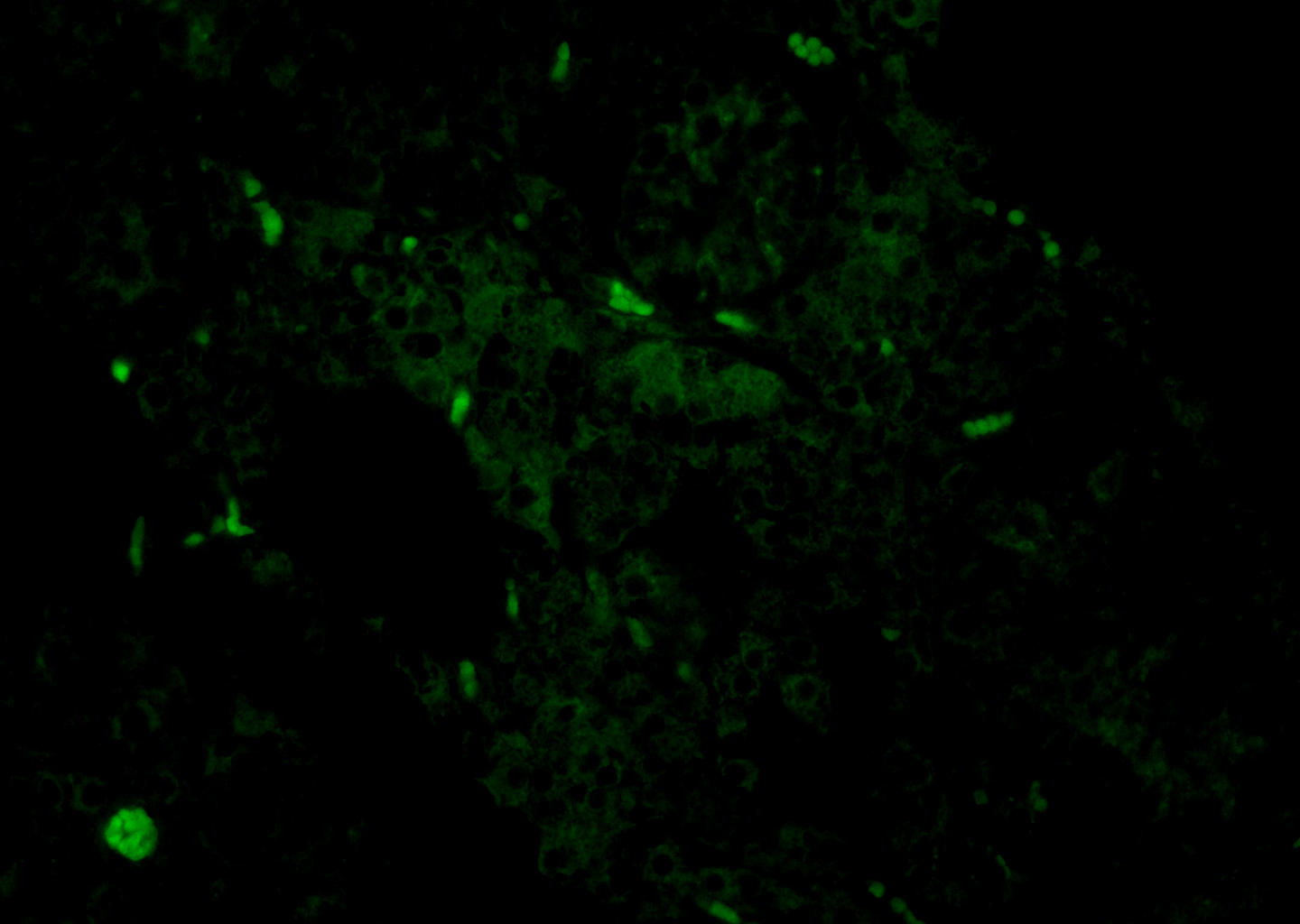

Supplement: Supplementary file 7 [file DataSheet4.ZIP › Immunofluorescence/6日睾丸原位杂交固定(小) SOX9红+DNMT39绿 400-8.tif]

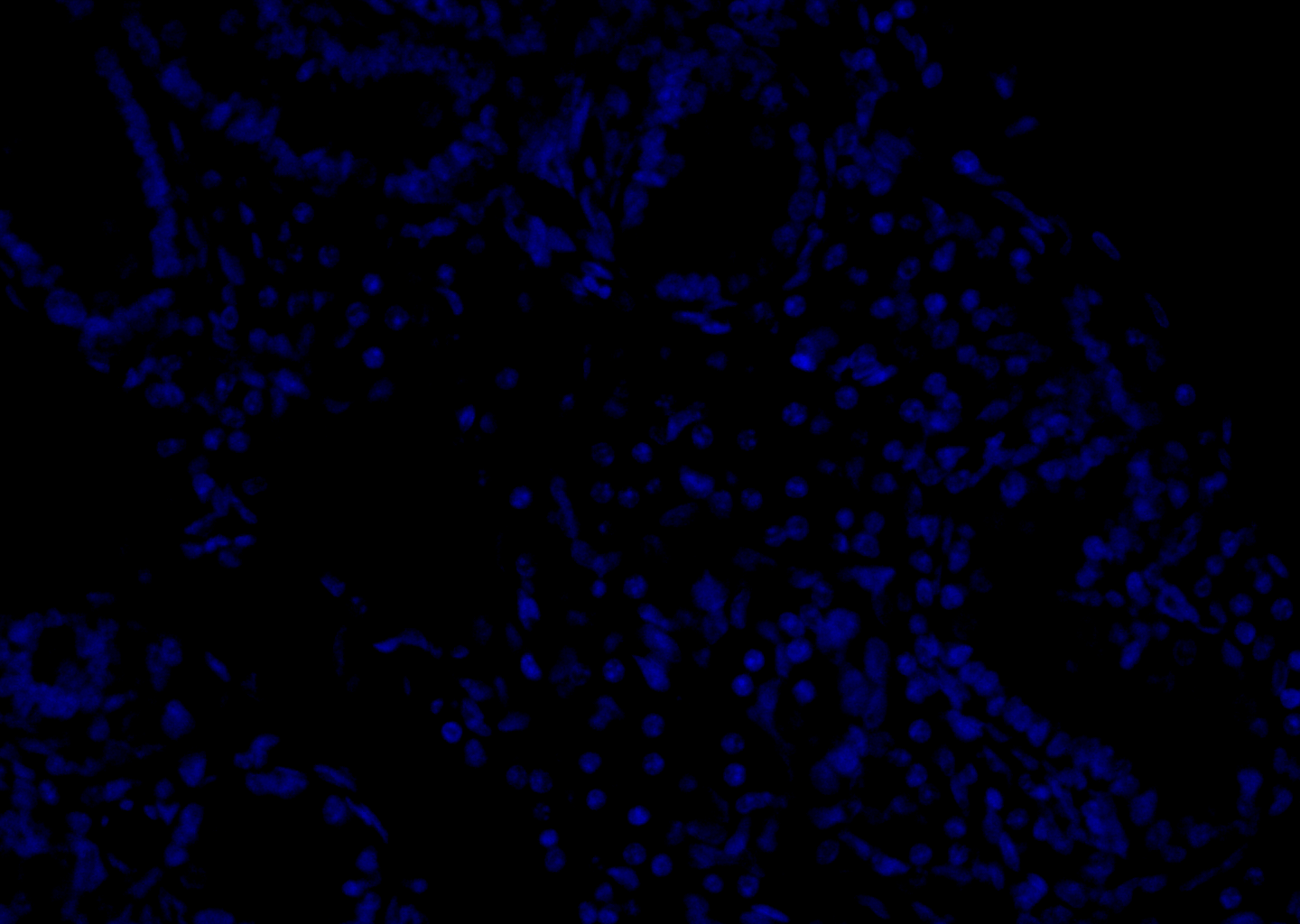

Supplement: Supplementary file 7 [file DataSheet4.ZIP › Immunofluorescence/6日睾丸原位杂交固定(小) SOX9红+DNMT39绿 400-9.tif]

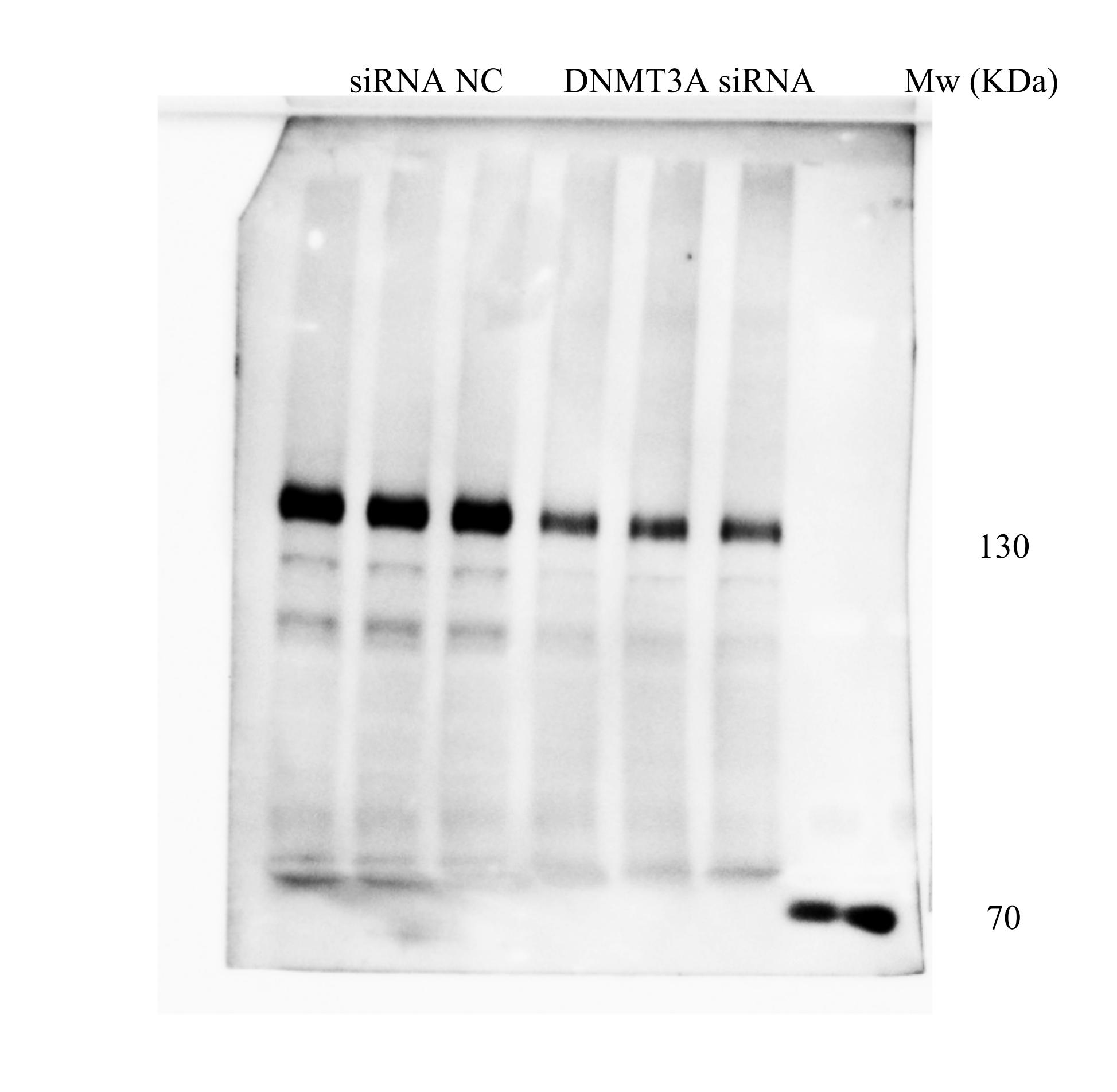

Supplement: Supplementary file 8 [file Image1.JPEG]

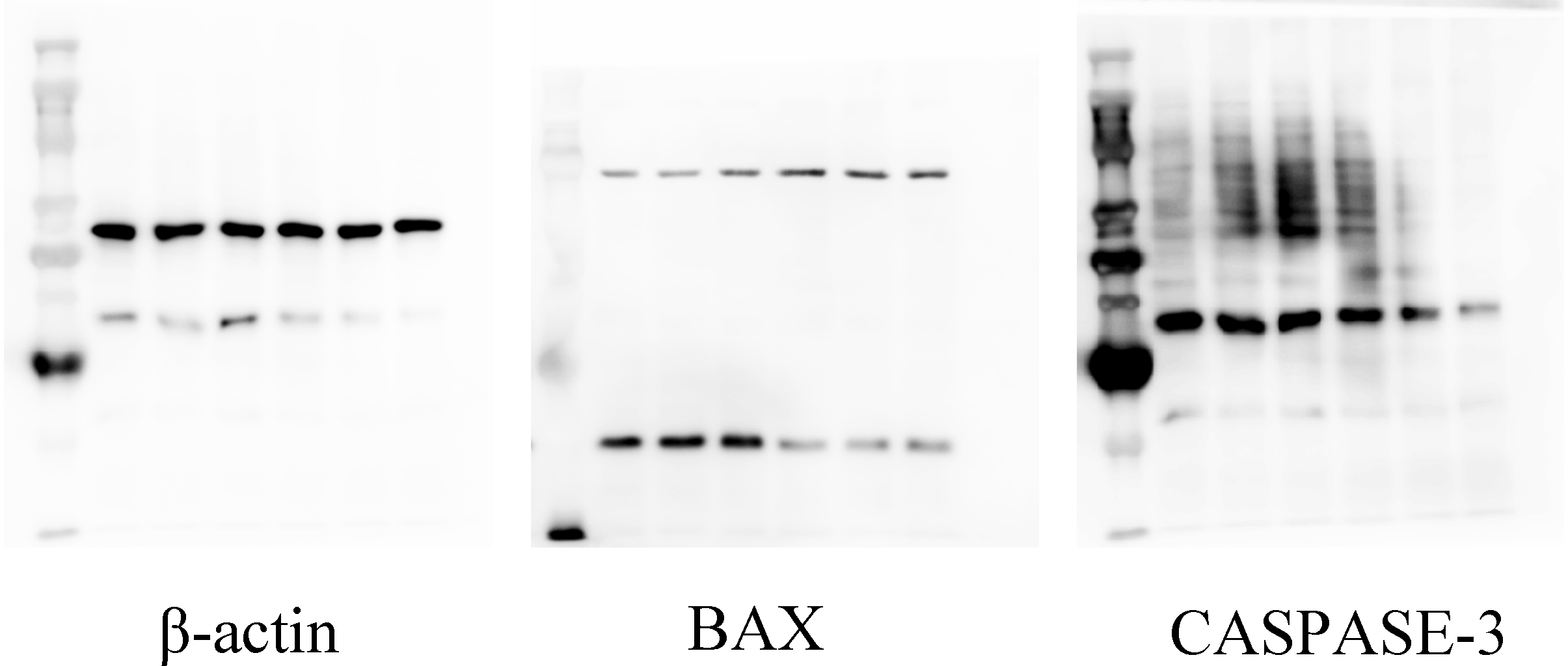

Supplement: Supplementary file 10 [file Image2.TIF]

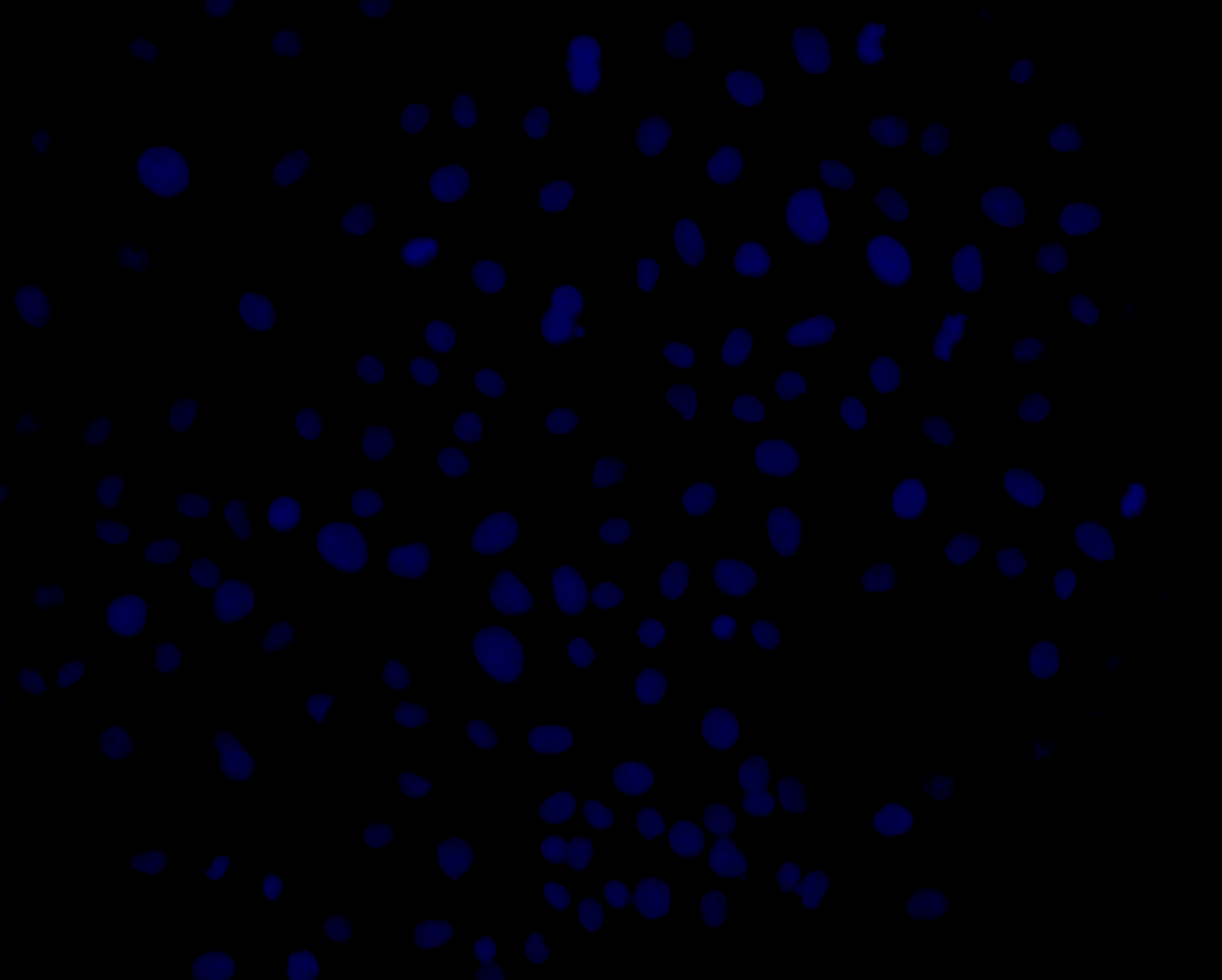

Supplement: Supplementary file 16 [file DataSheet5.ZIP › EdUmicroscopy images/拍摄-12744.tif]

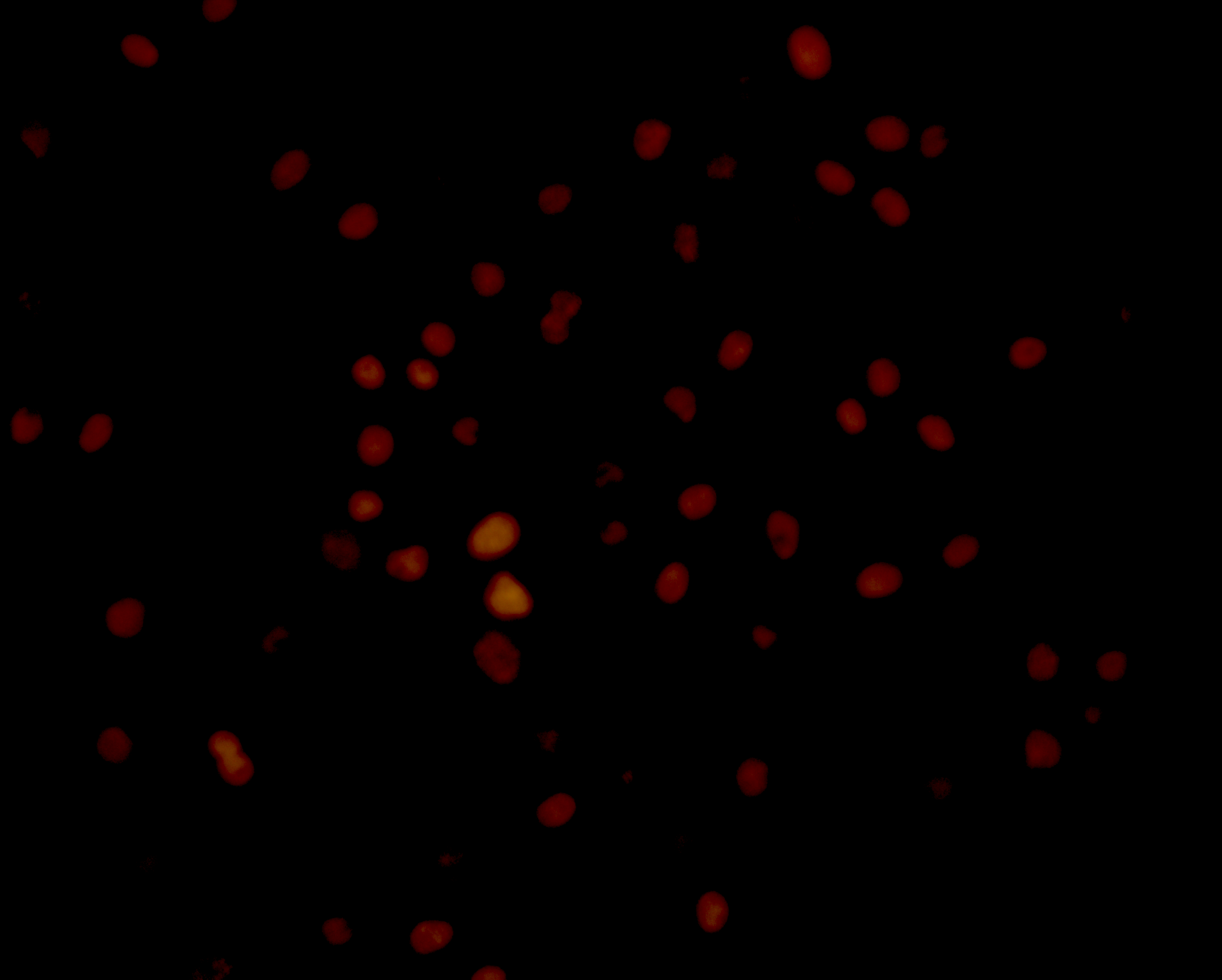

Supplement: Supplementary file 16 [file DataSheet5.ZIP › EdUmicroscopy images/拍摄-12745.tif]

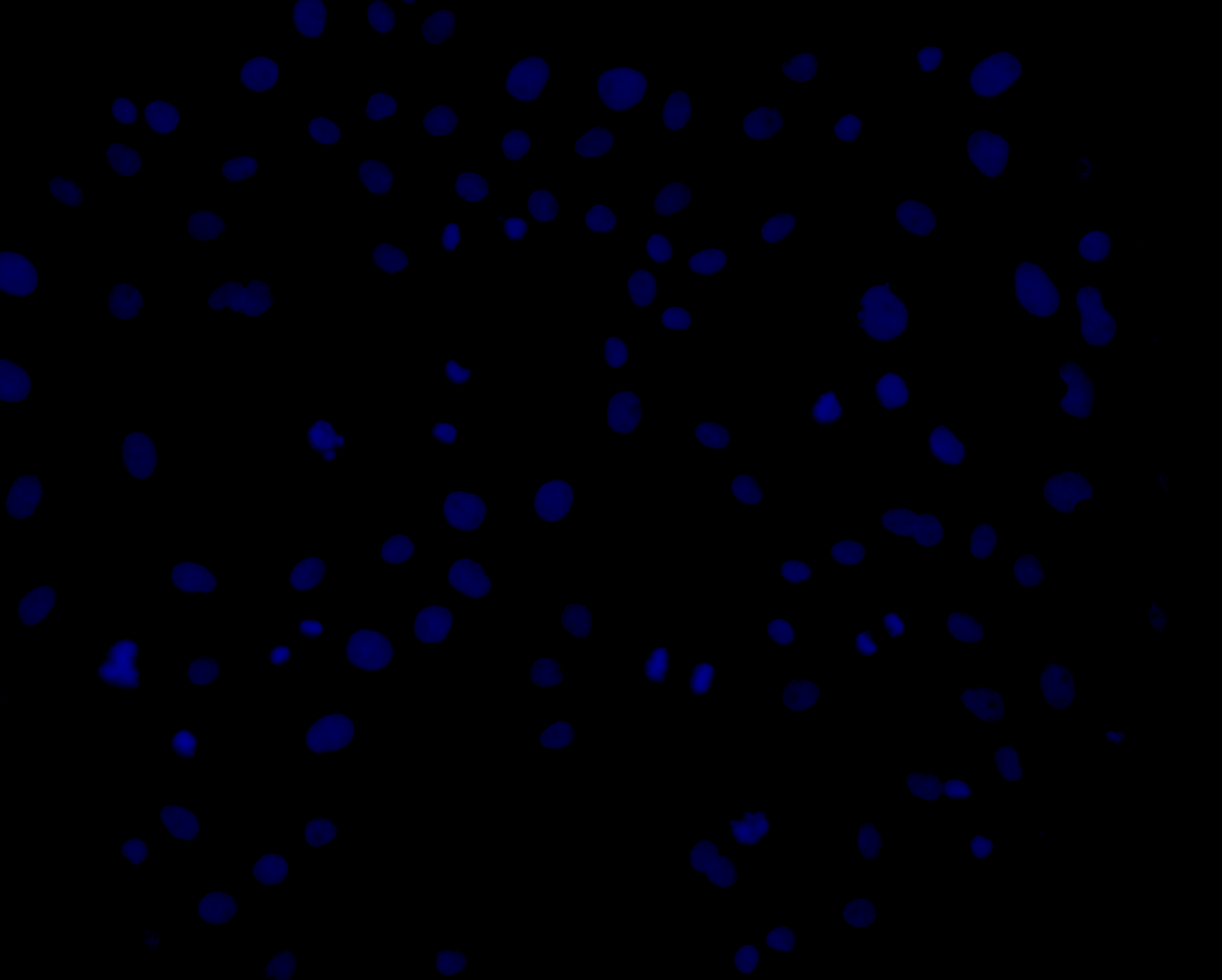

Supplement: Supplementary file 16 [file DataSheet5.ZIP › EdUmicroscopy images/拍摄-12792.tif]

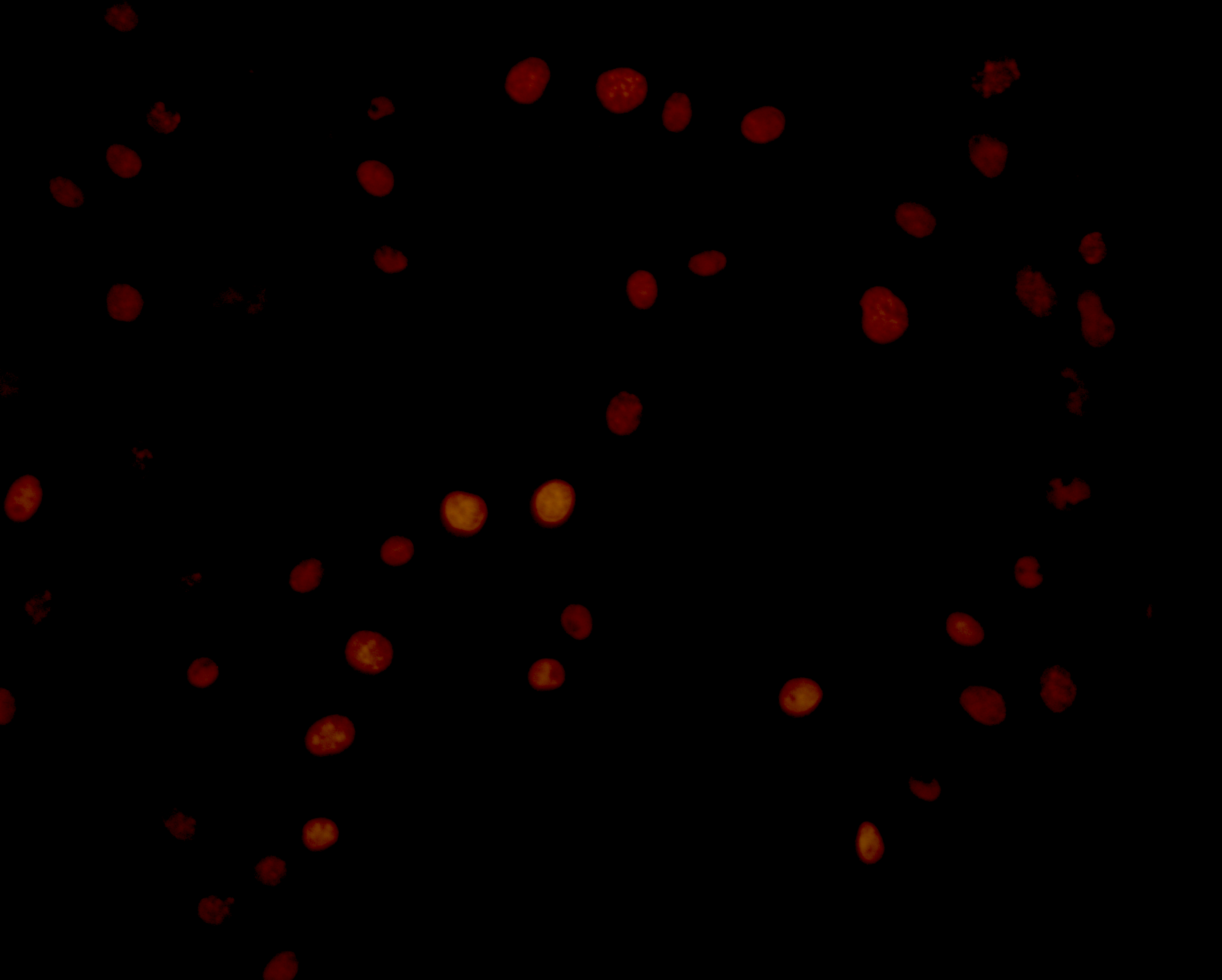

Supplement: Supplementary file 16 [file DataSheet5.ZIP › EdUmicroscopy images/拍摄-12793.png]

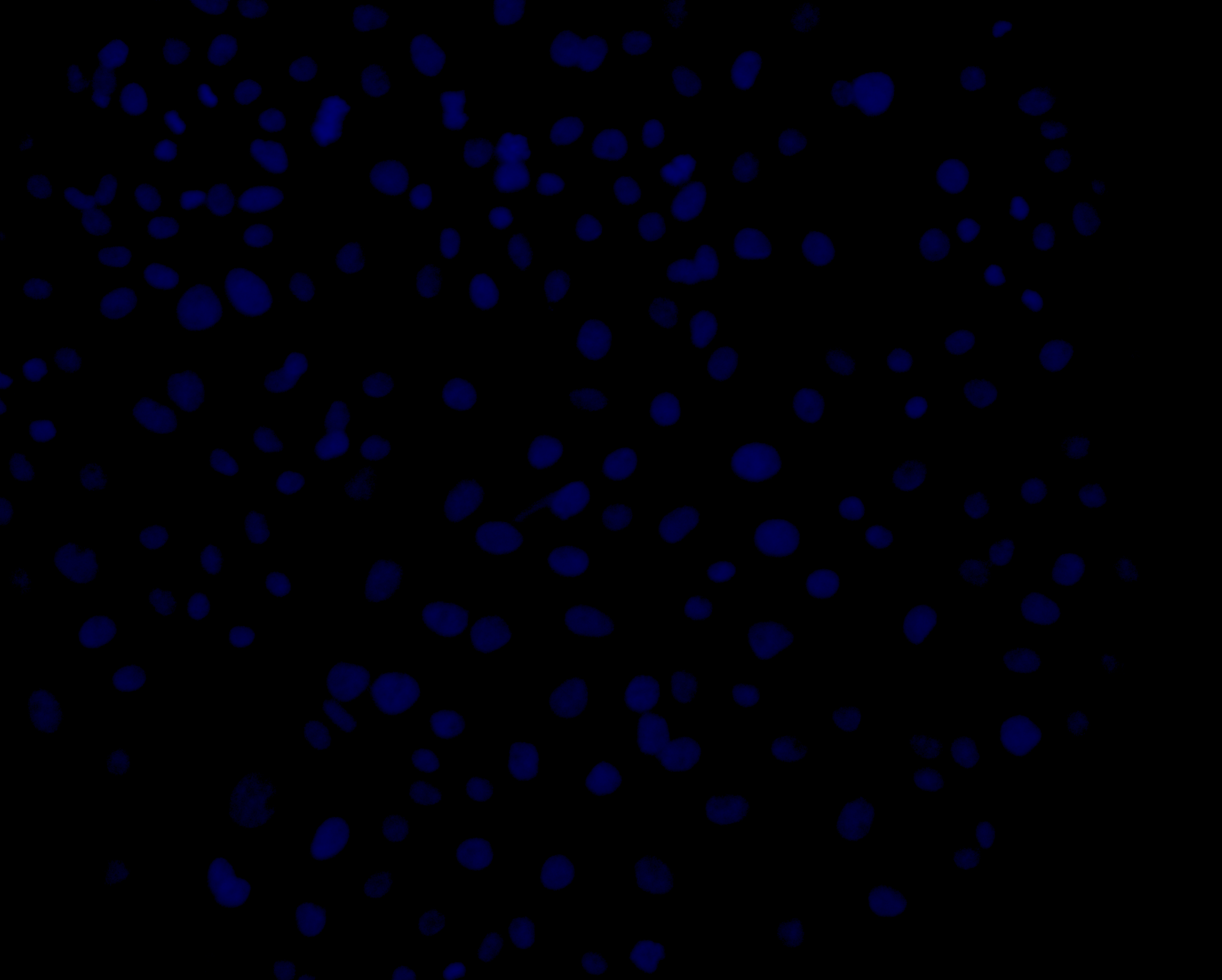

Supplement: Supplementary file 16 [file DataSheet5.ZIP › EdUmicroscopy images/拍摄-12843.tiff]

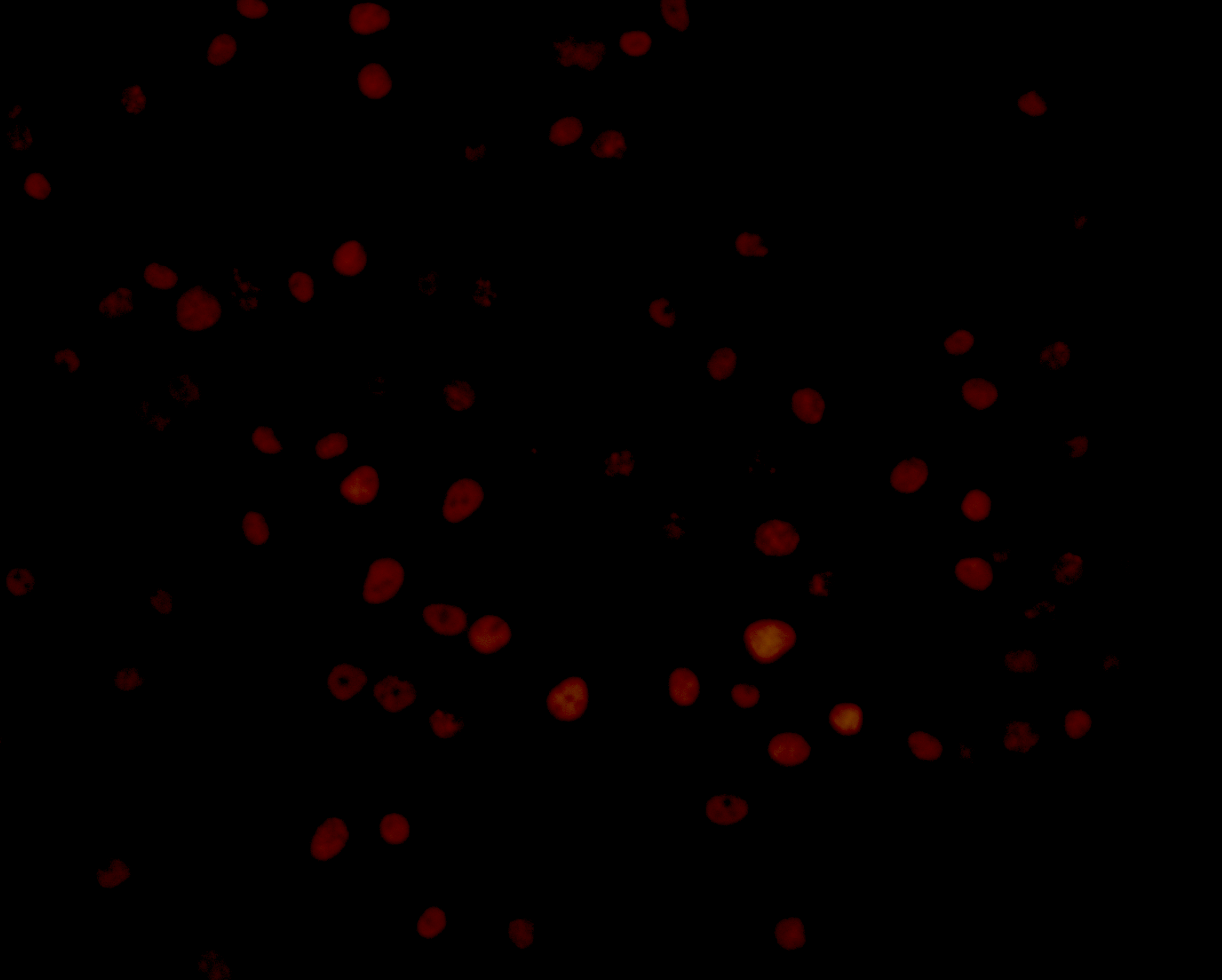

Supplement: Supplementary file 16 [file DataSheet5.ZIP › EdUmicroscopy images/拍摄-12844.tif]
