# Supplementary material for: Integration of RRBS and RNA-seq unravels the regulatory role of DNMT3A in porcine Sertoli cell proliferation
Source: Front Genet. 2024 Jan 9;14:1302351. doi: 10.3389/fgene.2023.1302351 (PMC10803568; doi:10.3389/fgene.2023.1302351)
Supplement: Supplementary file 13 [file DataSheet1.PDF]

```
* Create a dataset with two groups (Treat and NC), each with three repeated measures;
DATA mydata;
	INPUT Group $ Value;
	DATALINES;
Treat1
Treat2
Treat3
NC1
NC2
NC3
;
RUN;

* Use PROC STANDARD for Z-score standardization;
PROC STANDARD DATA=mydata OUT=zscoreData MEAN=0 STD=1;
	VAR Value;
	BY Group;
RUN;

* Use PROC PRINT to view the Z-score standardized data;
PROC PRINT DATA=zscoreData;
RUN;

* Use PROC TTEST for the t-test;
PROC TTEST DATA=zscoreData;
	CLASS Group;
	VAR Value;
RUN;
```
