# Supplementary material for: Integration of RRBS and RNA-seq unravels the regulatory role of DNMT3A in porcine Sertoli cell proliferation
Source: Front Genet. 2024 Jan 9;14:1302351. doi: 10.3389/fgene.2023.1302351 (PMC10803568; doi:10.3389/fgene.2023.1302351)

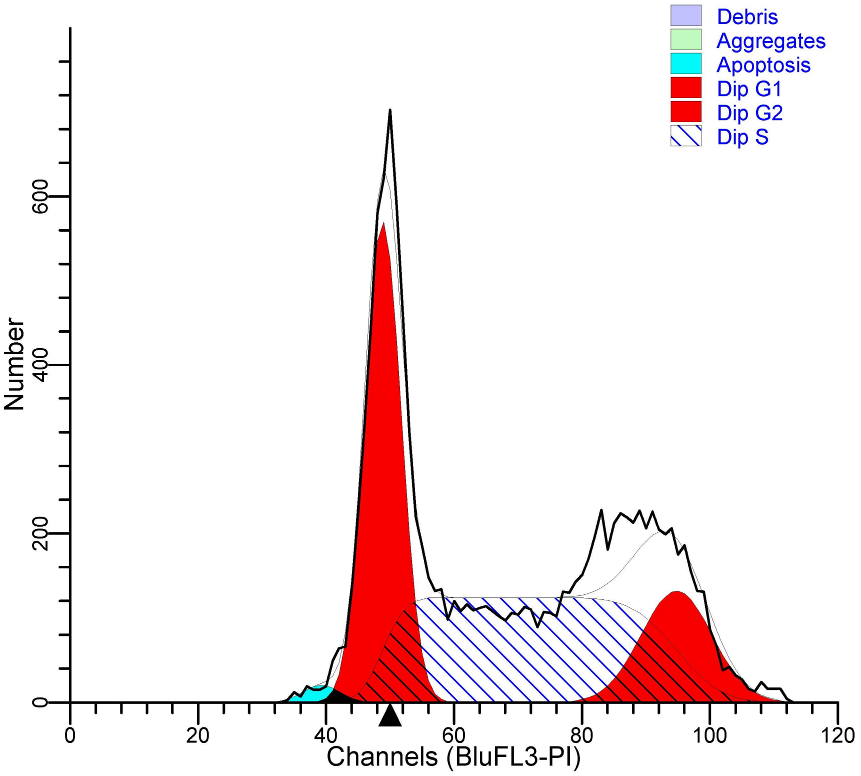

File analyzed: 20230524-DS-3-031.fcs
Date analyzed: 2-Jun-2023
Model: 1DA0A_DSD
Analysis type: Manual analysis

Ploidy Mode: First cycle is diploid

Diploid: 100.00 %
Dip G1: 35.28 % at 48.84
Dip G2: 15.76 % at 94.76
Dip S: 48.96 % G2/G1: 1.94
%CV: 5.85

Total S-Phase: 48.96 %
Total B.A.D.: 0.00 %

Apoptosis: 1.26 % Mean: 39.24

Debris: 0.00 %
Aggregates: 0.03 %
Modeled events: 11808
All cycle events: 11656
Cycle events per channel: 248
RCS: 3.494

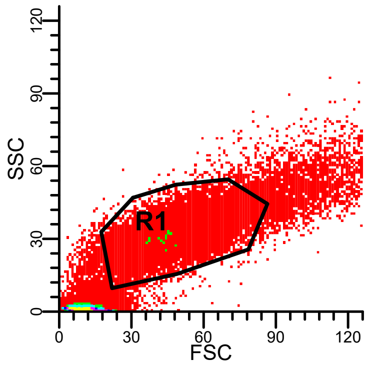

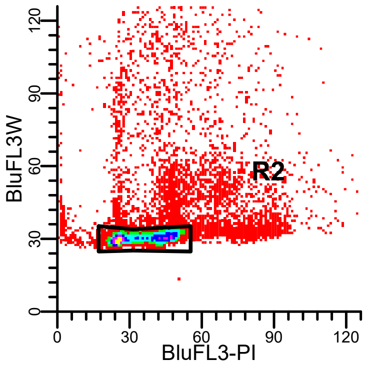

Supplement: Supplementary file 14 [file DataSheet2.ZIP › flow cytometry/cell cycle/DS-3.pdf]
