# Supplementary material for: Integration of RRBS and RNA-seq unravels the regulatory role of DNMT3A in porcine Sertoli cell proliferation
Source: Front Genet. 2024 Jan 9;14:1302351. doi: 10.3389/fgene.2023.1302351 (PMC10803568; doi:10.3389/fgene.2023.1302351)
Supplement: Supplementary file 14 [file DataSheet2.ZIP › flow cytometry/cell cycle/DS-4.pdf]

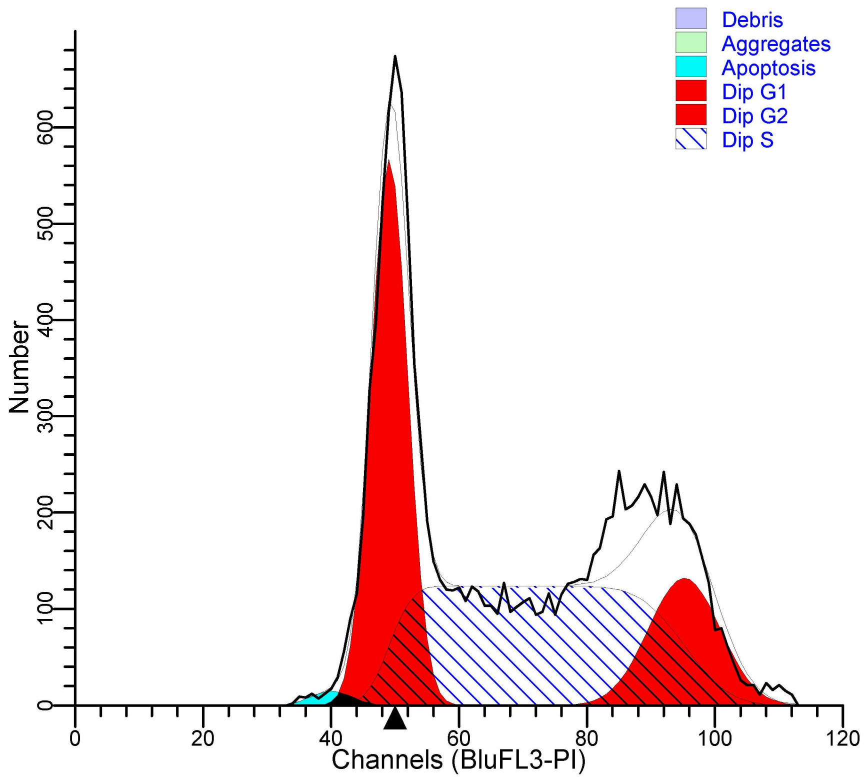


File analyzed: 20230524-DS-4-032.fcs
Date analyzed: 2-Jun-2023
Model: 1DA0A_DSD
Analysis type: Manual analysis

Ploidy Mode: First cycle is diploid

Diploid: 100.00 %
Dip G1: 35.16 % at 49.07
Dip G2: 15.83 % at 95.19
Dip S: 49.00 % G2/G1: 1.94
%CV: 5.85

Total S-Phase: 49.00 %
Total B.A.D.: 0.00 %

Apoptosis: 1.03 % Mean: 39.99

Debris: 0.00 %
Aggregates: 0.00 %
Modeled events: 11798
All cycle events: 11677
Cycle events per channel: 248
RCS: 3.533

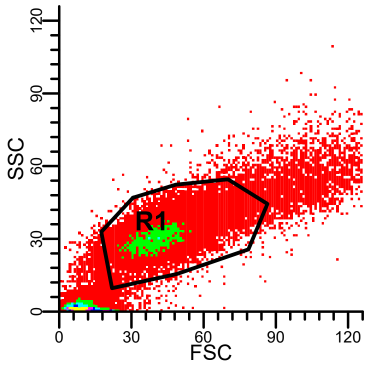


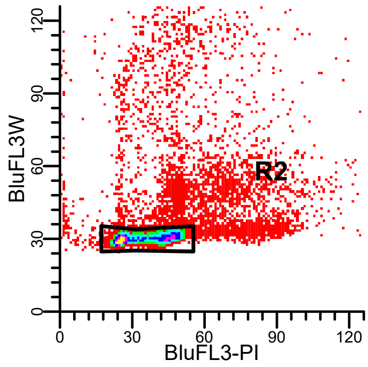


ModFitLT V3.1(Win32)
