# Supplementary material for: Integration of RRBS and RNA-seq unravels the regulatory role of DNMT3A in porcine Sertoli cell proliferation
Source: Front Genet. 2024 Jan 9;14:1302351. doi: 10.3389/fgene.2023.1302351 (PMC10803568; doi:10.3389/fgene.2023.1302351)
Supplement: Supplementary file 14 [file DataSheet2.ZIP › flow cytometry/cell cycle/DS-5.pdf]

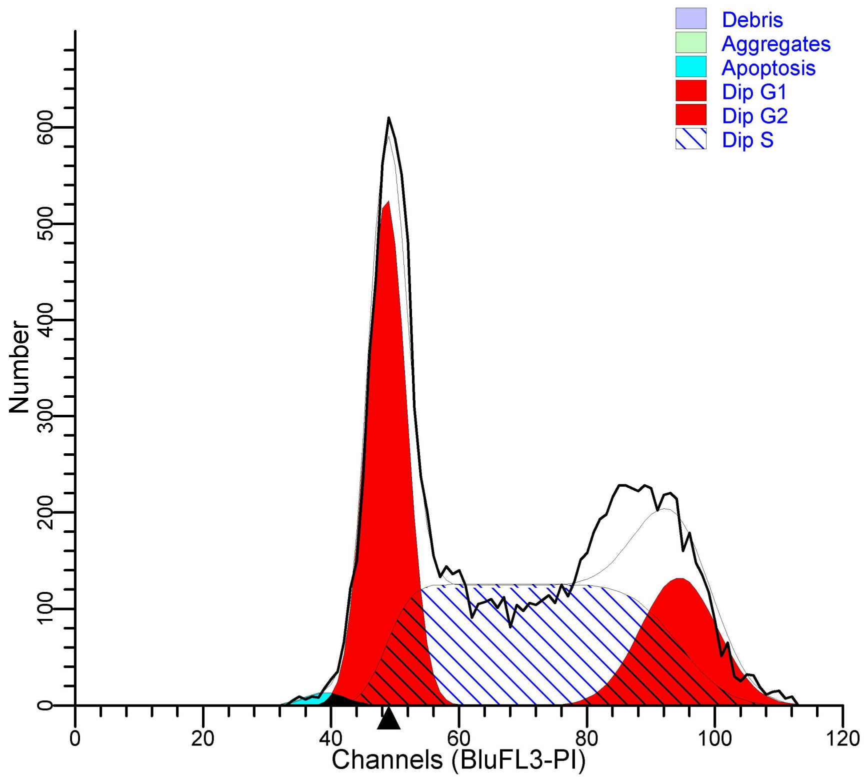

File analyzed: 20230524-DS-5-033.fcs
Date analyzed: 2-Jun-2023
Model: 1DA0A_DSD
Analysis type: Manual analysis

Ploidy Mode: First cycle is diploid

Diploid: 100.00 %
Dip G1: 34.64 % at 48.64
Dip G2: 16.84 % at 94.36
Dip S: 48.52 % G2/G1: 1.94
%CV: 6.35

Total S-Phase: 48.52 %
Total B.A.D.: 0.00 %

Apoptosis: 0.91 % Mean: 39.01

Debris: 0.00 %
Aggregates: 0.00 %
Modeled events: 11950
All cycle events: 11841
Cycle events per channel: 253
RCS: 3.541

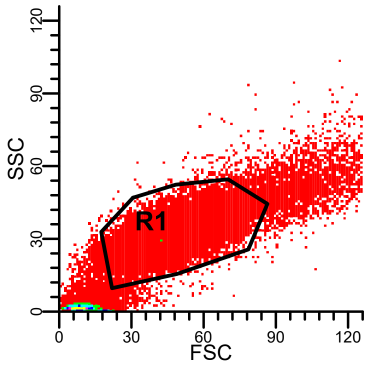

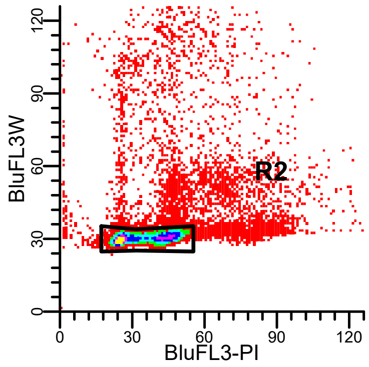

ModFitLT V3.1(Win32)
