# Supplementary material for: Integration of RRBS and RNA-seq unravels the regulatory role of DNMT3A in porcine Sertoli cell proliferation
Source: Front Genet. 2024 Jan 9;14:1302351. doi: 10.3389/fgene.2023.1302351 (PMC10803568; doi:10.3389/fgene.2023.1302351)

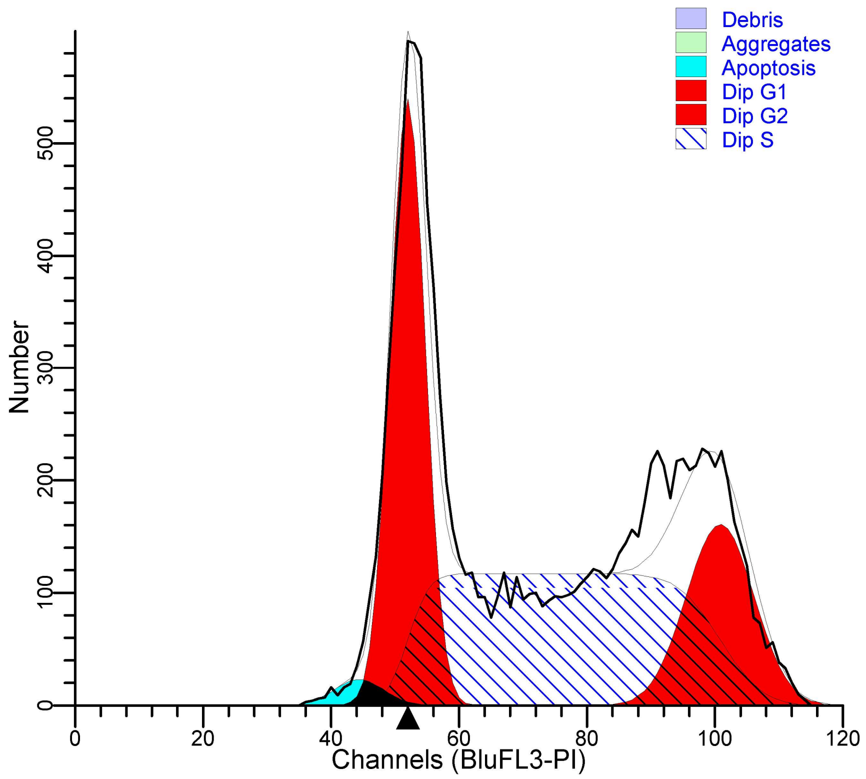

File analyzed: 20230524-N3-021
Date analyzed: 2-Jun-2023
Model: 1DA0A_DSD
Analysis type: Manual analysis

Ploidy Mode: First cycle is diploid

Diploid: 100.00 %
Dip G1: 32.06 % at 51.95
Dip G2: 18.51 % at 100.79
Dip S: 49.43 %   G2/G1: 1.94
%CV: 5.24

Total S-Phase: 49.43 %
Total B.A.D.: 0.00 %

Apoptosis: 1.89 %   Mean: 44.36

Debris: 0.00 %
Aggregates: 0.00 %
Modeled events: 11769
All cycle events: 11547
Cycle events per channel: 232
RCS: 4.007

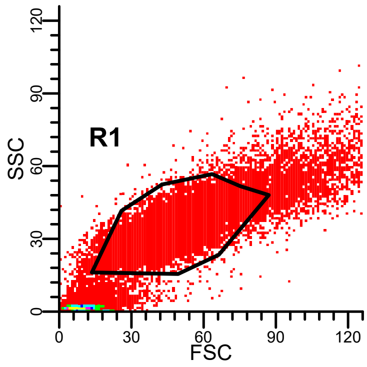

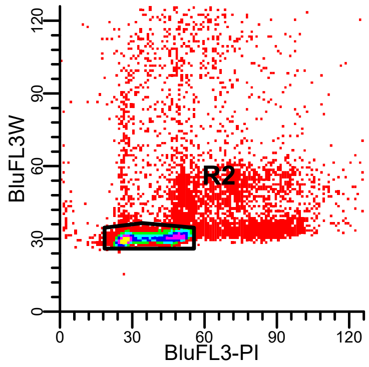

Supplement: Supplementary file 14 [file DataSheet2.ZIP › flow cytometry/cell cycle/N-3.pdf]
