# Supplementary material for: Integration of RRBS and RNA-seq unravels the regulatory role of DNMT3A in porcine Sertoli cell proliferation
Source: Front Genet. 2024 Jan 9;14:1302351. doi: 10.3389/fgene.2023.1302351 (PMC10803568; doi:10.3389/fgene.2023.1302351)
Supplement: Supplementary file 14 [file DataSheet2.ZIP › flow cytometry/cell cycle/N-5.pdf]

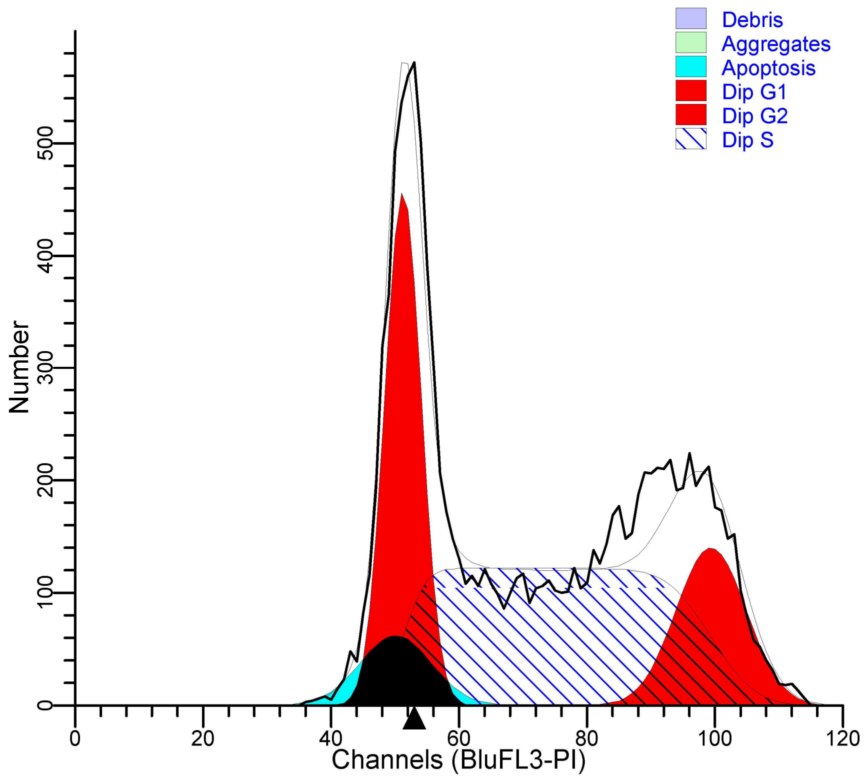

File analyzed: 20230524-N5-023
Date analyzed: 2-Jun-2023
Model: 1DA0A_DSD
Analysis type: Manual analysis

Ploidy Mode: First cycle is diploid

Diploid: 100.00 %
Dip G1: 29.47 % at 51.23
Dip G2: 17.46 % at 99.38
Dip S: 53.08 %   G2/G1: 1.94
%CV: 5.51

Total S-Phase: 53.08 %
Total B.A.D.: 0.00 %

Apoptosis: 7.31 %   Mean: 50.08

Debris: 0.00 %
Aggregates: 0.00 %
Modeled events: 11907
All cycle events: 11037
Cycle events per channel: 225
RCS: 2.858

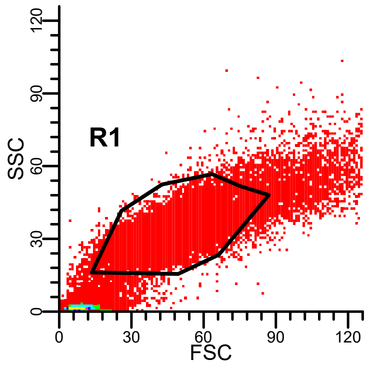

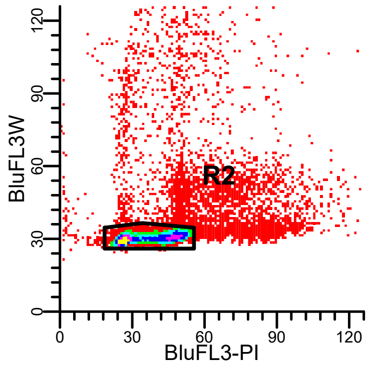

ModFitLT V3.1(Win32)
